# Geographical variation in morphology of *Chaetosiphella stipae stipae* Hille Ris Lambers, 1947 (Hemiptera: Aphididae: Chaitophorinae)

**DOI:** 10.1038/srep43988

**Published:** 2017-03-08

**Authors:** Karina Wieczorek, Agnieszka Bugaj-Nawrocka, Mariusz Kanturski, Gary L. Miller

**Affiliations:** 1 Department of Zoology, Faculty of Biology and Environmental Protection, University of Silesia in Katowice, Katowice, Poland; 2 Department of Zoology, Faculty of Biology and Environmental Protection, University of Silesia in Katowice, Katowice, Poland; 3 Department of Zoology, Faculty of Biology and Environmental Protection, University of Silesia in Katowice, Katowice, Poland; 4 United States Department of Agriculture (USDA), Agricultural Research Service, Systematic Entomology Laboratory, Beltsville, Maryland, USA

## Abstract

*Chaetosiphella stipae stipae* is a xerothermophilous aphid, associated with Palaearctic temperate steppe zones or dry mountain valleys, where there are grasses from the genus *Stipa*. Its geographical distribution shows several populations that are spread from Spain, across Europe and Asia Minor, to Mongolia and China. Geographical variation in chaetotaxy and other morphological features were the basis to consider whether individuals from different populations are still the same species. Moreover, using *Ch. stipae stipae* and *Stipa* species occurrences, as well as climatic variables, we predict potential geographical distributions of the aphid and its steppe habitat. Additionally, for *Stipa* species we projected current climatic conditions under four climate change scenarios for 2050 and 2070. While highly variable, our results of morphometric analysis demonstrates that all *Ch. stipae stipae* populations are one very variable subspecies. And in view of predicted climate change, we expect reduction of *Stipa* grasslands. The disappearance of these ecosystems could result in stronger separation of the East-European and Asian steppes as well as European ‘warm-stage’ refuges. Therefore, the geographic morphological variability that we see today in the aphid subspecies *Ch. stipae stipae* may in the future lead to speciation and creation of separate subspecies or species.

Genus *Chaetosiphella* Hille Ris Lambers, 1939 belongs to the tribe Siphini (Aphididae: Chaitophorinae) and includes six taxa (species and subspecies): *Ch. berlesei* (Del Guercio, 1905), *Ch. tshernavini* (Mordvilko, 1921), *Ch. stipae* subsp. *stipae* Hille Ris Lambers, 1947, *Ch. massagetica* Kadyrbekov, 2005, *Ch. longirostris* Wieczorek, 2008 and *Ch. stipae* subsp. *setosa* Wieczorek, 2008. Previous studies on this genus related mainly to descriptions of new morphs, distribution and host plants^1^,^2^,^3^,^4^,^5^,^6^,^7^,^8^,^9^. Here, we focus on *Ch. stipae stipae* which is a xerothermophilous aphid associated with Palaearctic temperate steppe zones or dry mountain valleys. Its distribution ranges from the Central Asia (including the basins and plateaus) and Ukrainian steppes to central and western Europe (Pannonian Basin and other isolated localities – including the dry inner Alpine valleys associated with xerothermic *Stipa* grasslands)^9^. *Ch. stipae stipae* live on the stems and upper side of leaves and are distinguished by their long apical rostral segment, long hind legs, and chaetotaxy. However, variations in chaetotaxy^9^ suggest that populations may have different taxonomies.

*Ch. stipae stipae* is a narrow oligophagous subspecies which requires very tight synchrony with its host plant phenology. It is mainly related to plants from the genus *Stipa* L. (Poaceae) – commonly called feather grass, needle grass, and spear grass. Depending on systematic treatment^10^, *Stipa* is comprised of 100 (www.efloras.org) to nearly 400 annual and perennial species (www.theplantlist.org). These grasses grow in dense, shorted clumps, and have no stolons. Leaves are stubbly with open leaf sheaths, and the inflorescence is a panicle with unifloral spikelets. *Stipa* spp. are mainly pollinated by wind and are facultatively cleistogamous, producing chasmogamous as well as cleistogamous flowers^11^. Many species of *Stipa* are protected, particularly in Europe^12^,^13^.

The Eurasian Steppe or Great Steppe, is the world’s largest steppe ecoregion where one of the dominant plant communities is *S. capillata* group^14^. Generally, the ecoregion extends from Eastern Europe (South Ukraine) to North China^15^ and is characterized by grassland plains without trees. However, grass and shrub steppes also occur as a narrow transition zone from Morocco in the West to Iran in the East^15^,^16^. In the north, the steppe borders the boreal forest (taiga) of central Russia and Siberia; in the south it transitions into desert^17^,^18^,^19^.

Grasslands are one of the most species-rich and diverse plant communities, and especially in Central and Western Europe, are very important refuges for xerothermic species of plants and invertebrates, including endemics^16^,^20^,^21^. Xerothermic grasslands, which are extra-zonal analogues of steppes, are among Europe’s most endangered natural environments^12^,^13^. Climatic and environmental conditions led to historical range changes in the distribution of vegetation and animals in Eurasia. During the Pleistocene, mid-latitudes of Eurasia were dominated by steppe-tundra habitat. These habitats have been shifting during interglacial intervals (mostly in the Holocene)^22^. Because of the regression of steppe-like habitats, steppes are now restricted to their refugial areas, particularly in Europe. So called ‘warm-stage’ refugium of steppe-like habitats^23^,^24^ are now restricted to Pannonian Basin^25^ and the Iberian Peninsula^26^. Isolated xerothermic grasslands located in the Balkans and central and western Europe could also be considered as cryptic ‘warm-stage’ refugias. These habitats are closely related to the continental steppes of Eurasia and are considered threatened due to anthropogenic transformation of the environment and the fact that they are isolated and limited (mainly to the areas unfavorable for forest plantations and agriculture).

As the first goal of this study we consider morphological variation of *Ch. stipae stipae* across geographical and environmental space via multivariate morphometric techniques. Aphid multivariate morphometric analyses (i.e. multiple discriminant analysis and the use of canonical varieties) have demonstrated the differences between closely related taxa or samples from clearly defined populations^27^,^28^,^29^,^30^. We also wanted to investigate how the habitat of this aphid might be affected by climate change and if geographic morphological variability of this subspecies may lead to environmentally-associated speciation in those populations. Therefore, the second goal of this study is use species distribution modeling of *Stipa* species to predict current *Ch. stipae stipae* distribution and predict how this distribution might be altered due to climate change.

## Results

### Statistical analysis of Taxon

**Figure 1:**
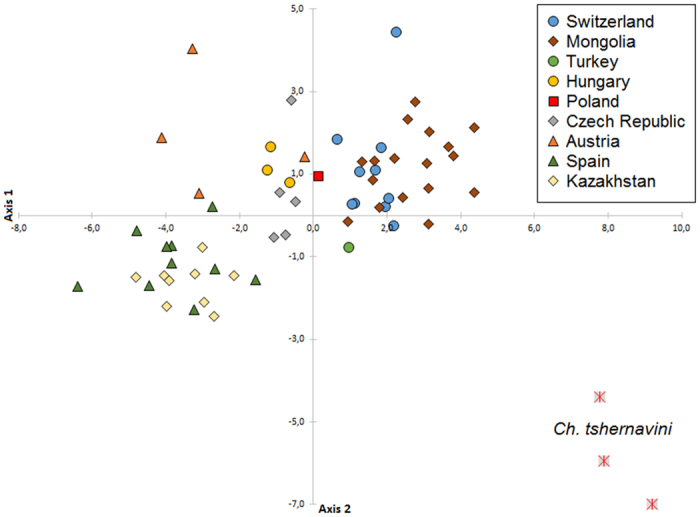
Canonical analysis of 61 specimens of *Chaetosiphella stipae stipae* (individuals divided according to the place of occurrence) and 3 specimens of *Ch. tshernavini* based on the analysis of 9 morphological variables and ratios; specimens projected onto the first and second principal axes.

**Figure 2:**
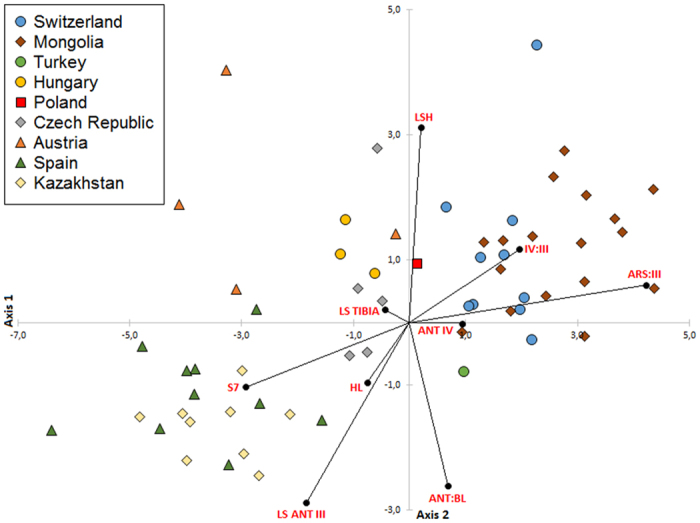
Canonical analysis of specimens of *Chaetosiphella stipae stipae* (individuals divided according to the place of occurrence) with the appointment of the impact of 9 morphometric variables and ratios; specimens projected onto the first and second principal axes.

Among the 16 morphometric variables and 7 morphometric ratios analyzed, 9 were selected based on the analysis of the correlation: ANT IV, LSH, HL, LS ANT III, LS TIBIA, S7, ARS:III, ANT:BL and IV:III. The first two axes of the CA represent 88.1% of the total variance (the first three axes represent 94.2%). The first component (axis 1) is characterized by the length of the longest seta on the seventh abdominal tergite (S7) and the ratio of ARS:III. The second component (axis 2) reflected the length of longest seta of head (LSH), length of longest seta of third antennal segment (LS ANT III) and the ratio of antennal lengths to body length (ANT:BL). Plotting CV1 and CV2 demonstrated no clear between-sample differentiation according to locality for *Ch. stipae stipae* (Figs 1 and 2). Therefore these results indicated that all studied individuals are most likely one morphologically variable subspecies. These morphological variations were reflected by the inconsistency of the number and the shape of abdominal setae for the individuals analyzed.

**Figure 3:**
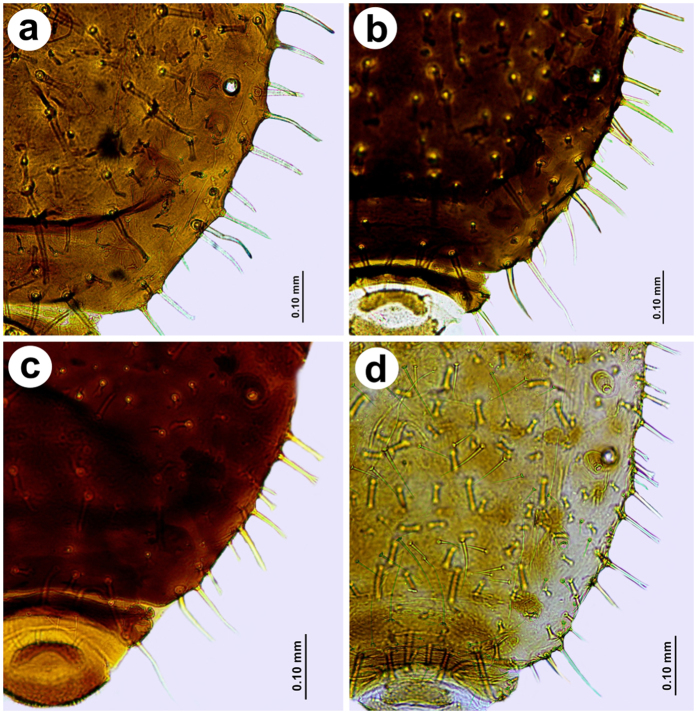
Abdominal chaetotaxy of representatives of *Chaetosiphella stipae stipae* from: (**a**) Austria, (**b**) Czech Republic, (**c**) Hungary, (**d**) Poland (typical set of abdominal chaetotaxy).

**Figure 4:**
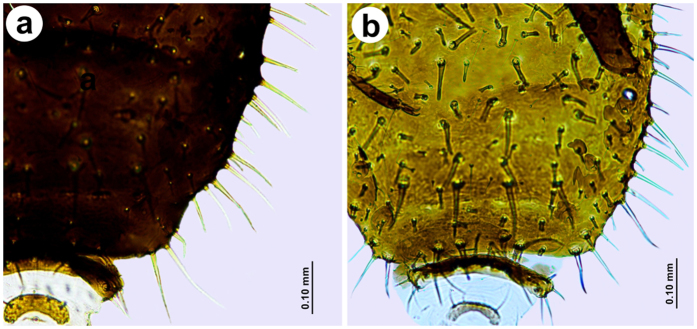
Abdominal chaetotaxy of representatives of *Chaetosiphella stipae stipae* from: (**a**) Spain, (**b**) Switzerland (individuals much “hairy”, with additional numerous long and pointed marginal setae on the tergites IV-VII).

**Figure 5:**
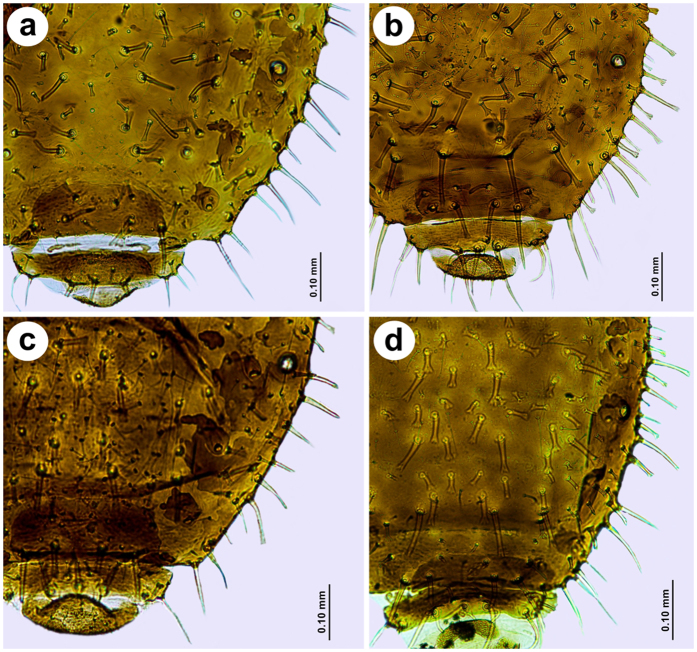
Abdominal chaetotaxy of representatives of *Chaetosiphella stipae stipae* from: (**a**) Kazakhstan, (**b**) Spain, (**c**) Mongolia, (**d**) Switzerland (typical set of abdominal chaetotaxy).

Individuals from central part of Europe (Austria, Czech Republic, Hungary, Poland (Fig. 3a–d) are characterized by having the typical set of abdominal chaetotaxy: marginal setae on abdominal tergites I-VI are 0.10–0.12 mm long and have forked and jagged apices; abdominal tergites VII and VIII have pointed setae 0.10–0.15 mm long. Pleural and spinal setae are 0.05–0.075 mm long, have forked and jagged apices interspaced with numerous, short, fan-shaped setae 0.025–0.03 mm long; the abdominal tergite VII has the longest, forked setae 0.10–0.12 mm long. Moreover, specimens from Turkey share some morphological features with specimens from central part of Europe (i.e. ARS, S7 or ARS:III) and Kazakhstan (i.e. LSH). Although some specimens from Swiss and Spanish populations are apparently different (Fig. 4a,b), morphometric analysis, shows they are within the range of variation. Specimens from Kazakh and Spanish populations share characters different than other populations studied: longer antennae, shorter apical segment of the rostrum, longer jagged marginal seta on the first abdominal segment, longer setae of hind tibia and the lowest ratio of the apical segment of the rostrum to the third antennal segment. Abdominal chaetotaxy is also similar (Fig. 5a,b), however some specimens from Spain are more setosae, with additional numerous long and pointed marginal setae on the abdominal tergites IV–VII (Fig. 4a), (these individuals are the most distant on the graph in the third quadrant (−, −)). Specimens from Mongolia (Fig. 5c) and Switzerland (Fig. 5d) populations share the following characters: shorter third antennal segment, shorter hind legs, similar length of the longest seta on the first abdominal tergite (S1), as well as the highest ratios of ANT V:III, ARS:ANT III and ARS:HT II. Similarly, some individuals from Switzerland are more setosae with additional numerous long and pointed marginal setae on abdominal tergites IV-VII (Fig. 4b), (these specimens are the most distant on the graph in the first quadrant (+, +)).

### Evaluation of the model and the importance of environmental predictors

The selection of settings for each model was chosen based on the results of pAUC, AICc and ΔAICc. In both models for *Ch. stipae stipae*, we observed better values of pAUC, AICc and ΔAICc were achieved where we used linear, quadratic and product (LQP) features together, regardless of the number of iterations. Otherwise it was with model for *Stipa* species – ΔAICc had the best value for models with auto features, but pAUC values were the best for models with LQP features. Most likely, this is due to the amount of the occurrence points used in the models. In both cases, we decided that we will use models with LQP features because models with auto features produced biologically nonsensical curves (jagged and not smooth).

Moreover, for *Ch. stipae stipae* models, the regularization multiplier performed better at a lower level than the default (0.5 and 0.75). Therefore, the areas predicted for the aphid generally corresponded to vegetation types where it is known to occur. For *Stipa* species model, the regularization multiplier performed the best result at value 1.25.

Two Maxent models were initiated for aphids and one for the *Stipa* species (one for present [Table t1][Table t2]climate projected to sixty-four for future climate scenarios). The average AUC values for the test data for *Ch. stipae stipae* are 0.863 (standard deviation = ±0.110 which is 11.0%) for the model based only on climate variables and 0.868 (SD =  ± 0.091, i.e. 9.1%) for the model based on climate variables plus the outcome model of potential distribution of host plants. For the *Stipa* species (present climate scenario), AUC was 0.878 (SD = ±0.03, i.e. 3.0%).

Because only four climatic variables were used for *Ch. stipae stipae* (see Table 3 for explanation which ones and why), the jackknife test (for details and other Maxent model outputs see Supplementary File S1) shows all of them were rather important (precipitation of warmest quarter (Bio18) was the least important). When the projected distribution of suitable habitat for *Stipa* species was included in model calibration, Maxent identified this variable as the most important, and affecting the reduction of areas potentially favorable for *Ch. stipae stipae*. In the case of *Stipa* species, the most important environmental variable was the mean temperature of warmest quarter (Bio10).

### Ecological niches and potential distribution

**Figure 6:**
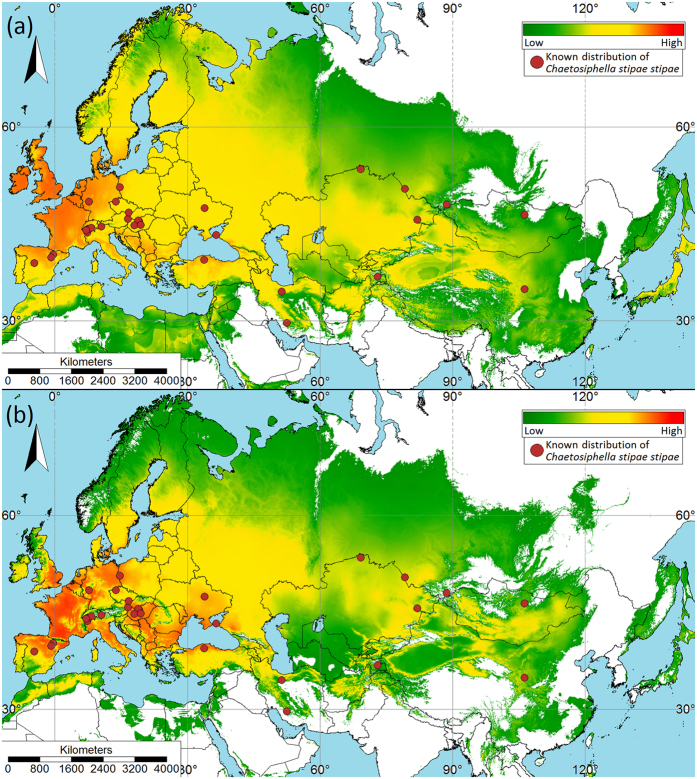
Maps of potentially suitable niches for *Chaetosiphella stipae stipae*. (**a**) Model based only on climate variables, and (**b**) model based on climate variables and outcome model of potential distribution of *Stipa* species. Circles represent known locations of aphids. Scale bars show Maxent logistic output (used for visualization purposes); higher values (warmer colours) indicate higher predicted suitability. The map was plotted using SAGA GIS 3.0.0^60^ (http://www.saga-gis.org); projection – World Mercator (EPSG: 3395).

**Figure 7:**
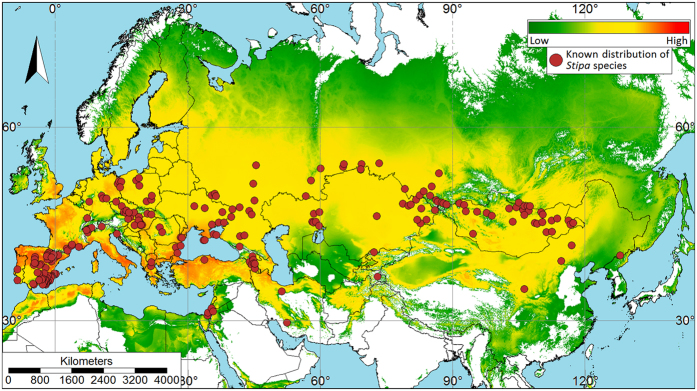
Maps of potentially suitable niches for representatives of the genus *Stipa*. Circles represent known locations of *Stipa* species. Scale bars show Maxent logistic output (used for visualization purposes); higher values (warmer colours) indicate higher predicted suitability. The map was plotted using SAGA GIS 3.0.0^60^ (http://www.saga-gis.org); projection – World Mercator (EPSG: 3395).

For both the aphid (Fig. 6a,b) and *Stipa* species (Fig. 7), maps of the median of the 10 model replicates were derived. The arithmetic mean was not used because it is not as resistant to outliers as the median (see Supplementary File S1 for plots showing distribution of occurrence records of *Ch. stipae stipae* and *Stipa* species in reference to used predictors). The logistic output was used. Therefore, the results range from 0 to 1.

The model suggested favorable climatic conditions for *Ch. stipae stipae* mainly in Europe. As potentially the most suitable areas in terms of climate, the model has appointed northern Spain, France, Belgium, the Netherlands, Germany, Denmark, the United Kingdom and Ireland. Other regions are Central Europe (excluding areas of the Alps and part of the Carpathians), Italy, Eastern Europe (excluding the areas of Russia), Turkey, the Balkans (excluding Dinaric Alps). In the Mediterranean region, such conditions occur in Morocco and the northern part of Algeria. In Asia, the model suggests suitable regions in the Caucasus, in certain regions of Iran, Afghanistan, Pakistan, Kyrgyzstan, Kazakhstan, and in foothill areas of north-western China (excluding the Tibetan Plateau and Quiling Mountain, and the Tarim Basin, Dzungarian Basin and Turfan Depression) (Fig. 6a).

When the projected distribution of *Stipa* species was included as a biotic variable in the model for *Ch. stipae stipae*, the distribution of suitable habitat for the aphid was projected as more constrained. Primarily, range has been limited in the north and south of Europe as well as in Asia. Mountainous areas (e.g., the Alps, Carpathian, Pyrenees and Caucasus Mountains) have also been eliminated (Fig. 6b). Supplementary Table S1 details ecoregions which are occupied by *Ch. stipae stipae*. These are mainly regions within the temperate broadleaf and mixed forests, temperate conifer forests, temperate grasslands, savannas and shrublands, Mediterranean forests, woodlands and scrub, and montane grasslands and shrublands.

In addition, according to the model, the species of grasses of the genus *Stipa* will find suitable conditions in Europe similar to those of the aphid (albeit slightly broader), but in Asia the area is definitely broader. The model suggested areas typical for these grasses in the Pontic-Caspian steppe, Central and Eastern Anatolian steppe in Turkey, Kazakh steppe and forest steppe, Emin Valley steppe, Mongolian-Manchurian grassland and Daurian forest steppe. These areas extend from Moldova, Romania and Ukraine, by Russia and Kazakhstan, to Mongolia and China.

### Climatic preferences

Possible climatic preferences of aphid as well as *Stipa* species were inferred by comparing potential ecological niches to the Köppen-Geiger climate classification. The analysis of climate data from known localities of *Ch. stipae stipae* shows that it prefers places with an average monthly temperature above 10 °C (50 °F) in warmest months (April to September). Such conditions are characteristic for continental climates like warm summer continental climates (Dfb, Dwb) in central and eastern Europe and Russia, as well as Mediterranean (Csb) (Spain) and oceanic (Cfb) climates in Spain and Germany. Some individuals were located in the steppe type of climate associated with cold semi-arid climate (BSk) in Spain, or even cold desert climate (BWk) on the border of Russia and Mongolia. However, individuals from the population in Switzerland and Italy, which was located in the Alps, apparently have other preferences. A mountain or highland climate in this area is characterized by absence of a month with a mean temperature higher than 10 °C (50 °F). However, the locations of aphids are in the lower parts of the mountains – in the valleys, where the temperature is slightly higher.

Grasses of the genus *Stipa* have a similar climate preference to *Ch. stipae stipae* which feed on them. However, besides the aforementioned preference on warm summer subtype of a continental climate, *Stipa* species preferred additional areas include interior Eurasia, east-central Asia, and parts of India.

### Comparison of current and future suitable habitat for *Stipa* species habitat

Suitable climatic conditions for *Stipa* steppes habitat were predicted to decline under all four RCP scenarios by the 2050 s (Supplementary File S1 Fig. 1.16a–d and 1.18a–d) and the 2070 s (Supplementary File S1 Fig. 1.17a–d and 1.19a–d). At present time, 32,471,127 km^2^ were designated as suitable in Europe, Asia and North Africa (with 10^th^ percentile training presence threshold). For the 2050 climate scenarios, the amount of suitable habitat could decrease to 81.9–87.9% of the area in common with the current model (it decrease to 63.3–75.2% for the higher threshold) (Table 1). For the 2070 climate scenarios, those amounts could decrease to 75.6–88.1% of the area in common with the current model (46.1–73.5% for the higher threshold). In both versions of the future climate scenarios, the higher the concentration of carbon dioxide, the more mean annual temperature increased (Supplementary File S1 Fig. 1.20a,b). Therefore, generally our results indicated that the potential distribution range of steppes habitat with *Stipa* species will be reduced over time.

## Discussion

*Ch. stipae stipae* is an example of a strictly xerothermophilous subspecies originating in the East-European and West-Asian steppe. It is also encountered in European ‘warm-stage’ refuges of steppe-like habitats. Similar to its host plants (*Stipa* spp.), the existing populations of *Ch. stipae stipae* are highly isolated from each other. However, based on the results of our morphometric analysis, these populations all represent one highly variable aphid subspecies, *Ch. stipae stipae. Stipa* grasslands developing on limestone bedrock are exposed to high-amplitude variation in temperature, both on a daily and annual basis. Those local conditions (e.g., in the dry inner Alpine valleys), possibly influence morphological characters and lead to multiplication of abdominal chaetotaxy in some individuals of *Ch. stipae stipae*. This confirms the presence of closely related *Ch. stipae setosa* presently known only from a few localities in Alpes-Côte d’Azur Provence, France. *Ch. stipae setosa* can be distinguished from nominal taxon by the length and form of apical segment of rostrum (much shorter) and numerous, pointed marginal setae. The marginal setae with forked or jagged apices, which are diagnostic character of *Ch. stipae stipae*, are absent^8^.

Models confirm favorable climatic conditions in most of areas where the presence of individuals representing the studied subspecies has already been recorded^9^ (see Supplementary File S1 for detailed maps). In Asia, suitable regions have been suggested by the model in foothill areas of the Caucasus, Altay Mts., Pamir Mts., Turbagatay submountain region and in north-western China (Mt. Xiaowutaishan). In Europe, this subspecies also prefers dry valleys at the foothills of the Alps and Pyrenees. When projected habitat suitability of *Stipa* was included as a predictor variable, suitable habitat for *Ch. stipae stipae* did not encompass the Alps and the Carpathians. However, on the enlarged map (Supplementary File S1 Fig. 1.9) we can see that suitable habitat was projected in mountain valleys. It should be noted that scale plays an important role in the modeling^31^,^32^,^33^. Therefore, we modeled the Eurasian area using a resolution of 2 km. But modeling on such a scale will negatively affect areas of refugia. In our example, it affected on warm mountain valleys that create the appropriate microclimate conditions for *Stipa* species and associated with them *Ch. stipae stipae*. By using a larger scale in the modeling, the result probably would be even more accurate.

Furthermore, according to the model, France, Belgium, the Netherlands and the United Kingdom should be suitable areas for *Ch. stipae stipae*, but this subspecies has not been recorded in those regions of Europe. Milder and warmer oceanic climate, conditions in these localities, may be appropriate for this aphid subspecies if the host plant condition is suitable. Unlike other aphids, *Ch. stipae stipae* do not form large and numerous colonies. Instead, they are cryptic and feed individually on stems and the upper side of the leaves. Because of this, their observation and collection, as with most of the tribe Siphini, is difficult and infrequent. There may be more species awaiting discovery in this tribe which would expand what is currently defined as their geographical range. For example, the recently described species *Atheroides vallescaldera* Miller and Jensen, 2014 (Siphini) from the USA, belongs to a genus previously considered exclusively Palaearctic in origin^34^. The use of ecological-niche models could be used as a convenient tool to predict both potential new species habitats and localities.

It is known that climate change affects various organisms. Both temperature and precipitation are important abiotic factors, which specifically affect plant life cycles, growth and their ranges of occurrence^35^. Such factors consequentially change interspecific relationships and plant community structure^36^,^37^. There are numerous studies regarding the influence of climate change on different types of vegetation in different regions of the world (e.g. European flora in general^38^) or just European forest ecosystems^39^, *Aloe* tree, Namib Desert^40^, temperate steppe in Inner Mongolia, China^41^ or agriculture in Africa and Latin America^42^. Climate change and its effects on terrestrial insects and herbivory patterns was reviewed by Cornelissen^43^. Predicting the potential impact of climate change on species distributions via ecological niche modeling represents a useful tool. And as noted by Sanchez *et al*.^44^, such models could be used by conservation practitioners, order to help them in the management of vegetation under the climate change. However, we should remember that apart from climatic changes, other abiotic parameters (e.g., land transformations or soil) also affect the habitat preferences and adaptation possibilities^45^. Unfortunately, due to lack of such data for the present study area, we were not able to include those parameters in the current analysis.

Each of the conducted scenarios in our study suggest that steppe habitat with representatives of the genus *Stipa* will shrink rather than expand into new areas. *Stipa* spp. are a C_3_ grasses, and similar like other C_3_ plants^33^, are characterized by the tendency to reduce abundance with increasing mean annual temperature^46^. Part of the areas that are now climatically suitable, according to data from raster used in the modeling, over time will become too warm and too dry. This applies in Europe to areas of Spain, but in particular to areas of Asia: southern Russia, Kazakhstan, Turkey, Azerbaijan, Iran, as well as China and Mongolia. Among the areas that will remain stable is Europe (excluding Spain) as well as a central part of Mongolia. Areas most at risk are the eastern part of the Pontic–Caspian steppe, Kazakh steppe and Emin Valley steppe – one of the most common Eurasian steppe ecoregions. The disappearance of these ecosystems could result in stronger separation of the East-European and Asian steppes as well as European ‘warm-stage’ refuges. There is the possibility of greater speciation events among both plants as well as insects associated with this environment. Therefore, the geographic morphological variability that we see today in the aphid subspecies *Ch. stipae stipae* may in the future lead to speciation and creation of separate subspecies or species^47^,^48^,^49^,^50^,^51^. The isolation of habitats may also promote this mechanism^52^,^53^.

## Methods

### Taxon sampling and statistical analysis

Sixty-one microscope slide-mounted specimens of *Ch. stipae stipae* were examined (three slide-mounted specimens of *Ch. tshernavini* Mordv. were also examined as an out group) from the local populations distributed in Spain (UL coll.), Switzerland (BMNH, RMNH coll.), Austria (BMNH coll.), Czech Republic (BMNH coll.), Poland (UŚ coll.), Hungary (ZMPA coll.), Turkey (MNHN coll.), Kazakhstan (USNM coll.) and Mongolia (ZMPA coll.). The slides were examined using a Nikon^®^ Ni-U light microscope and photographed with a Nikon^®^ DS-Fi2 camera. Measurements are given in mm (see Supplementary Table S2). The following abbreviations were used (partly following^54^): BL – body length (from anterior border of the head to the end of cauda); ANT – antennae or their lengths; ANT I, II, III, IV, V, VI – lengths of antennal segments I, II, III, IV, V, VI (ratios between antennal segments are simply given as e.g. ‘VI:III’); BASE – basal part of last antennal segment or its length; PT – processus terminalis of last antennal segment or its length; ARS – apical segment of rostrum or its length; HL – length of hind leg; HT II – second segment of hind tarsus or its length; LSH – length of longest seta of head; LS ANT III – length of longest seta of third antennal segment; BD ANT III – basal articular diameter of ANT III; S1 – the length of the longest seta on the first tergite; S7 – the length of the longest seta on the seventh tergite; LS TIBIA – length of longest seta of hind tibia.

Specimens were borrowed from the following scientific collections (preceded by acronyms used in this paper): MNHN – Muséum national d’Histoire naturelle, Paris, France; BMNH – the Natural History Museum, London, UK; UL – University of Leon, Leon, Spain; RMNH – Nationaal Natuurhistorisch Museum, Leiden, The Netherlands; USNM – U.S. National Museum of Natural History Aphid collection, located at the Henry A. Wallace Beltsville Agricultural Research Center, Beltsville, Maryland, USA; UŚ – entomology collection of the Department of Zoology, University of Silesia, Katowice, Poland; ZMPA – Museum and Institute of Zoology of the Polish Academy of Sciences, Warsaw, Poland.

Twenty-three morphometric variables and ratios were analyzed with the use of STATISTICA (StatSoft Inc, Tulsa, Oklahoma, USA) (Supplementary Table S2). The discriminant analysis module, applying stepwise discriminant function analysis (DFA), followed by canonical analysis (CA) was performed. Canonical analysis was used to determine variables which contributed most to separation of the locality-based groups.

### Occurrence data

Thirty-two unique occurrence localities were compiled for the *Ch. stipae stipae*; 188 occurrence localities were selected within the steppe ecoregions to include known host plants of the described aphid – species of the genus *Stipa (S. capillata, S. dasyphylla, S. joannis, S. kirghisorum, S. pennata* subsp. *eriocaulis, S. sibirica, S. splendens*). All occurrence data for the aphid were based on the detailed review of specimens studied in the museum collections and scientific literature (see abbreviations for depositories and literature references in Supplementary Table S1). Occurrence data for *Stipa* species were obtained from the GBIF database (www.gbif.org). Records with unknown or unspecified localities were not used. A Geographic Distance Matrix Generator 1.2.3 was used to calculate the geographic distance between each pair of localities^55^,^56^. Points close to each other less than 20 km have been removed – this distance was chosen to reduce the inherent geographic biases (effect of spatial autocorrelation) which are associated with methods of sample collecting. All localities were geo-referenced using Google Earth 7.1.2.2041^57^ (coordinates for localities were collected in decimal degrees, datum: WGS84).

Distribution data for individuals of *Ch. stipae stipae* subspecies will be published in the Global Biodiversity Information Facility (GBIF) database. For details of all occurrence localities for *Ch. stipae stipae* and *Stipa* species used in the MaxEnt model refer to Supplementary Table S1 and S3.

### Environmental predictors and climate classification

The standard nineteen bioclimatic variables, as well as future climate data (downscaled CMIP_5_ data) were downloaded from WorldClim 1.4 dataset (http://www.worldclim.org)^58^. Coupled Model Intercomparison Project Phase 5 (CMIP_5_) evaluates global warming based on global climate models, and one of the variables studied by these models is the climate sensitivity to changes in the concentration of carbon dioxide in various scenarios change^59^. To estimate the influence of global climate change on the potential distribution of *Stipa* species, the species distribution for three different time periods (present, 2050, and 2070) and for four future representative concentration pathways (RCPs) (+2.6, +4.5, +6.0 and +8.5 W/m^2^) were modeled. For future climate scenarios, the map of the average values were presented from eight CMIP_5_ model outputs: BCC-CSM1-1, CCSM4, GISS-E2-R, HadGEM2-AO, HadGEM2-ES, IPSL-CM5A-LR, MRI-CGCM3 and NorESM1-M. A spatial resolution of 60 arc-seconds (approximately 2 km^2^) for models was chosen (30 arc-seconds spatial resolution grids downloaded from WorldClim were interpolated to 60 arc-seconds spatial resolution). All maps were prepared in SAGA GIS 3.0.0 (http://www.saga-gis.org)^60^ using WGS84 datum and EPSG: 3395 (World Mercator). To calculate the area occupied by *Stipa* species, Lambert azimuthal equal-area projection was used^61^.

The Köppen-Geiger climate classification system^62^ was used to define climate preferences of *Ch. stipae stipae* and *Stipa* species. Raw climate classification data were extracted from raster layer at *Ch. stipae stipae* and *Stipa* occurrence records. Additionally, the resulting raster from Maxent were imposed on raster of Köppen-Geiger climate classification using SAGA GIS.

### Ecological niche modeling

In our study we present two models for aphids – one based only on climate variables, and the second additionally contains the result of modeling for *Stipa* species, included as a biotic variable. Models of the current distribution of *Ch. stipae stipae* as well as current and future (under climate change scenarios for 2050 and 2070) potential distribution of *Stipa* species were made using the Maxent software (version 3.3.3k; http://www.cs.princeton.edu/~schapire/maxent), which is based on a maximum entropy algorithm^63^. Ecological niche modeling was used to discern ecological aspects of *Ch. stipae stipae* and its habitat represented by *Stipa* species. This modeling tool has been widely used in faunal and floral studies and in many aspects of biology including ecology, evolutionary biology and biogeography^64^,^65^,^66^. The primary task of modeling is to predict the range of distribution of a species or plant community. This is based, in simple approach, on identifying areas with environmental conditions that will allow species to survive^67^,^68^,^69^.

By using SAGA GIS 3.0.0, raw environmental data were extracted from bioclimatic raster layers at *Ch. stipae stipae* and *Stipa* occurrence records, as well as from 10 000 background points from the entire study area (Europe and Asia). Through the use of Spearman rank correlation, performed in the R software (version 3.3.1)^70^ using Rattle package (version 3.5.0)^71^, the number of variables was minimized by discarding those which were highly correlated (*r* > 0.75 or *r* < −0.75). Variables that did not have any significant contribution to the model (did not match the habitat preferences of surveyed species) and were highly correlated, were removed. Table 2 presents selected variables and their association with the habitat preferences of *Ch. stipae stipae* and *Stipa* species.

As species occurrence points came from museum data and were not collected randomly, bias files were provided during Maxent modeling. Both bias grid files were generated in SAGA GIS, weighted by a Gaussian kernel with a standard deviation (SD) of 200 km (for instruction see ref. 72).

Many authors points out that the default settings in Maxent do not always produce the best predictions^73^,^74^,^75^,^76^. Therefore we used different regularization multiplier values (ranging from 0.5 to 2.5) and different combinations of feature types (auto features or linear, quadratic and product features together (LQP)). A 10-fold cross-validation was ran in both models – for aphid and for *Stipa* species^63^,^77^,^78^. A jackknife test was selected to show relative importance of each predictor by comparing models with all environmental variable combinations with individual variable importance.

As a threshold-independent assessment of overall model performance we used area under the ROC (receiver‐operating characteristic) curve (AUC), partial AUC (pAUC) (calculated using NicheA^79^) and the sample-size corrected Akaike’s information criterion (AICc and ΔAICc) (calculated using ENMTools 1.4.4^80^) (see Supplementary Table S4 for results). Calculation of AICc requires both the ‘.asc’ file and the .‘lambdas’ file associated with each model, and it is crucial for output to be in “raw” format.

We also employed threshold-dependent measure (omission rate based on threshold rule) –10^th^ percentile training presence threshold. It is set at a value that excludes the 10% of calibration localities with lowest prediction, so it has an expected omission rate of 0.10^81^. As there is still no consensus as to which threshold is the best, the 10^th^ percentile threshold has been more commonly used^82^,^83^,^84^. It is considered to provide a more ecologically significant result^85^,^86^. Moreover, other thresholds often require both presence and absence data, while 10^th^ percentile threshold is widely used when true absence data is not available^87^. Therefore, in order to convert from the continuous to binary maps and to define habitat and non-habitat areas, we used 10^th^ percentile training presence threshold for *Ch. stipae stipae* and *Stipa* species^77^,^88^. We also checked what changes will occur in the study area under the climate changes at the 50^th^ percentile training presence threshold. We did it because we wanted to know how much vulnerable to climate change are areas with a higher probability of suitable conditions.

The logistic output of Maxent with prediction values from 0 (unsuitable habitat) to 1 (optimal habitat) was used for final models. Table 3 presents settings that were finally chosen for all models.

## Additional Information

**How to cite this article:** Wieczorek, K. *et al*. Geographical variation in morphology of *Chaetosiphella stipae stipae* Hille Ris Lambers, 1947 (Hemiptera: Aphididae: Chaitophorinae). *Sci. Rep.* **7**, 43988; doi: 10.1038/srep43988 (2017).

**Publisher's note:** Springer Nature remains neutral with regard to jurisdictional claims in published maps and institutional affiliations.

## Supplementary Material

Supplementary Information

Supplementary Table S1

Supplementary Table S2

Supplementary Table S3

Supplementary Table S4

## Figures and Tables

**Table 1 t1:** Total area predicted to have probability of suitable habitat conditions for steppe habitat under climate change scenarios.

Scenario	Area (km^2^)	% change in area	Area common to current (km^2^) (stable)	% of area common to current
	>10^th^ percentile training presence threshold			
Current	32 471 127			
2050 s – RCP2.6	31 506 728	−3.0%	28 567 726	87.9%
2050 s – RCP4.5	31 248 402	−3.7%	27 616 076	85.0%
2050 s – RCP6.0	31 397 886	−3.3%	28 169 976	86.7%
2050 s – RCP8.5	30 896 024	−4.8%	26 609 548	81.9%
2070 s – RCP2.6	31 609 158	−2.6%	28 621 858	88.1%
2070 s – RCP4.5	30 986 949	−4.6%	26 830 320	82.6%
2070 s – RCP6.0	31 007 950	−4.6%	26 829 426	82.6%
2070 s – RCP8.5	30 293 509	−6.7%	24 556 589	75.6%
	>50^th^ percentile training presence threshold			
**Current**	10 602 773			
2050 s – RCP2.6	9 575 408	−9.7%	7 968 652	75.2%
2050 s – RCP4.5	9 700 688	−8.5%	8 055 047	86.0%
2050 s – RCP6.0	9 411 320	−11.3%	7 186 026	67.8%
2050 s – RCP8.5	9 199 519	−13.2%	6 711 345	63.3%
2070 s – RCP2.6	9 962 849	−6.0%	7 798 233	73.5%
2070 s – RCP4.5	9 386 385	−11.5%	6 773 825	63.9%
2070 s – RCP6.0	9 131 118	−13.9%	6 544 394	61.7%
2070 s – RCP8.5	8 278 156	−21.9%	4 884 476	46.1%

The results are presented for the two thresholds.

**Table 2 t2:** Bioclimatic variables chosen for modeling and their association with the habitat preferences of *Ch. stipae stipae* and *Stipa* species.

	Variable	Description	Rationale
*Chaetosiphella stiape stipae*	Bio01	Annual Mean Temperature [°C]	This variable approximates the total energy inputs for an ecosystem^89^. As individuals of the *Ch. stipae stipae* inhabit various steppe ecosystems (typical temperate steppe and Alpine-steppe), we want to know what temperature ranges are optimal.
	Bio10	Mean Temperature of Warmest Quarter [°C] – mean temperatures during the warmest three months of the year (in this case – June, July, and August)	As *Ch. stipae stipae* is considered a strictly xerothermophilous subspecies^90^, temperature and precipitation during the warmest period of the year are important for its habitat. This period is also critical for aphids because day length, temperature, and nutrition are recognized as the most important factors which influence the production of sexuales (oviparous females and males) in autumn^91^.
	Bio15	Precipitation Seasonality [mm] – a measure of the variation in monthly precipitation totals during the year or averaged years	As with variable Bio01, we wanted to know what range of precipitation is optimal for the occurrence of this aphid.
	Bio18	Precipitation of Warmest Quarter [mm]	See the description of the variable Bio10.
*Stipa* species – steppe habitat	Bio04	Temperature seasonality [°C] – a measure of temperature change during the year or averaged years	This variable was included in the analyses because steppes exhibit a wide variation of temperature, both during the year and the day
	Bio10	Mean Temperature of Warmest Quarter [°C] – mean temperatures during the warmest three months of the year (in this case – June, July, and August)	Since the Miocene, dry grasslands are linked to the warm and dry climate^92^, temperature and precipitation during the warmest period of the year seems very important for this type of vegetation. Warm summers combined with low precipitation generally reduce the presence of trees thus allowing the development of grass vegetation.
	Bio14	Precipitation of Driest Month [mm]	For the Great Steppe, driest months occur in the winter (mostly February). Winter precipitation penetrates deeper into the soil. Conversely, summer precipitation evaporates before infiltration^93^.
	Bio15	Precipitation Seasonality [mm] – a measure of the variation in monthly precipitation totals during the year or averaged years	Steppes are characterized by low precipitation – we want to see of precipitation variability during the year.
	Bio18	Precipitation of Warmest Quarter [mm]	See the description of variable Bio10.

**Table 3:** Settings selected for the Maxent models based on the results of evaluation methods.

Settings	*Chaetosiphella stipae stipae* (only climate variables)	*Chaetosiphella stipae stipae* (climate variables with output model for host plants)	*Stipa* species
maximum number of iterations	1000	500	1000
maximum number of background points	50000	50000	50000
regularization multiplier	0.5	0.75	1.25
convergence threshold	0.00001	0.00001	0.00001
feature type	LQP*	LQP	LQP

*LQP – linear, quadratic and product features together.
