# Supplementary material for: Geographical variation in morphology of Chaetosiphella stipae stipae Hille Ris Lambers, 1947 (Hemiptera: Aphididae: Chaitophorinae)
Source: Sci Rep. 2017 Mar 8;7:43988. doi: 10.1038/srep43988 (PMC5341063; doi:10.1038/srep43988)
Supplement: Supplementary Information [file srep43988-s1.pdf]

**Supplementary Information**

# Geographical variation in morphology of *Chaetosiphella stipae stipae* Hille Ris Lambers, 1947 (Hemiptera: Aphididae: Chaitophorinae)

Scientific Reports

Karina Wieczorek[1], Agnieszka Bugaj-Nawrocka[2]*, Mariusz Kanturski[3], Gary L. Miller[4]

[1,2,3] Department of Zoology, Faculty of Biology and Environmental Protection, University of Silesia, Katowice, Poland

[4] United States Department of Agriculture (USDA), Agricultural Research Service, Systematic Entomology Laboratory, Beltsville, Maryland, USA

*Correspondence: Agnieszka Bugaj-Nawrocka, Department of Zoology, Faculty of Biology and Environmental Protection, University of Silesia, Bankowa 9, 40-007 Katowice, Poland.

E-mail: abugaj-nawrocka@us.edu.pl

**Supplementary File S1: Maxent model outputs and locations of aphids and grasses records in the multidimensional climatic space. Additionally, detailed maps of potentially suitable niches for *Cheatosiphella stipae stipae* and *Stipa* species, as wella as predicted habitat for *Stipa* species under all four RCP scenarios for 2050s and 2070s.**

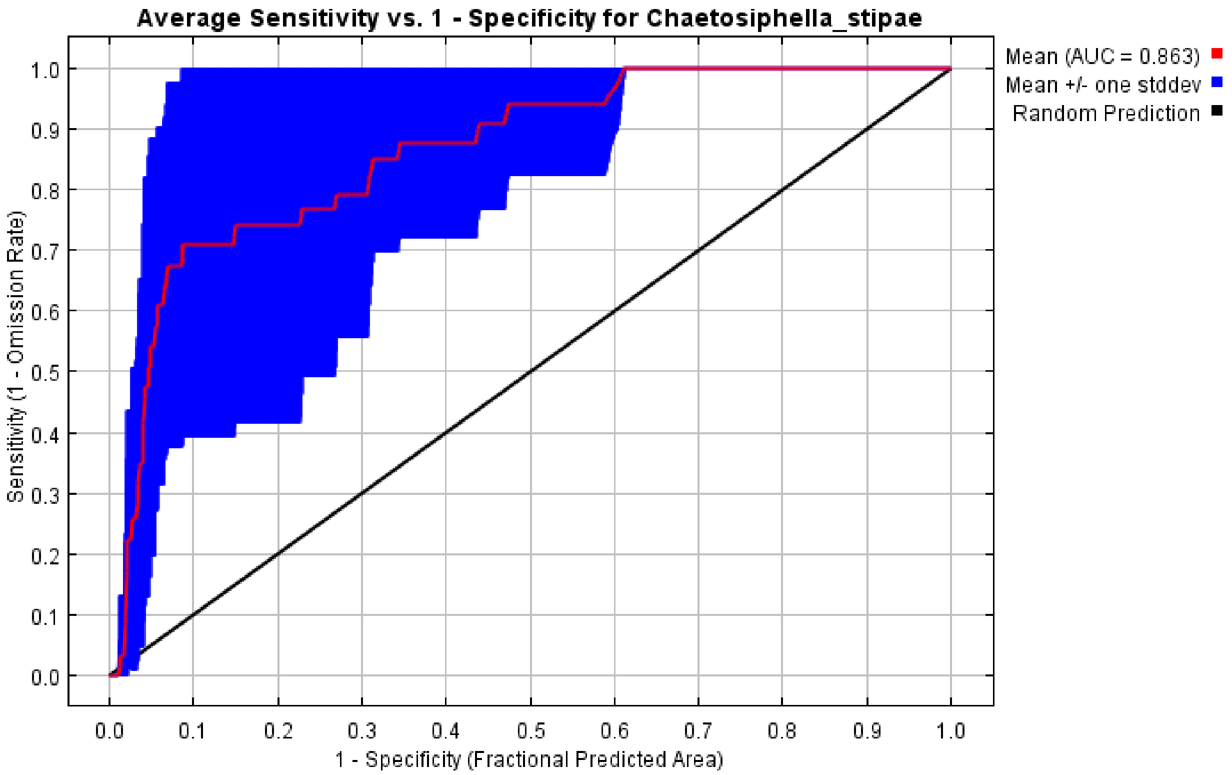


**Figure S1.1.** The receiver operating characteristic (ROC) curve generated in Maxent, showing an average of 10 repetitions of the model for *Chaetosiphella stipae stipae* (only climate variables).

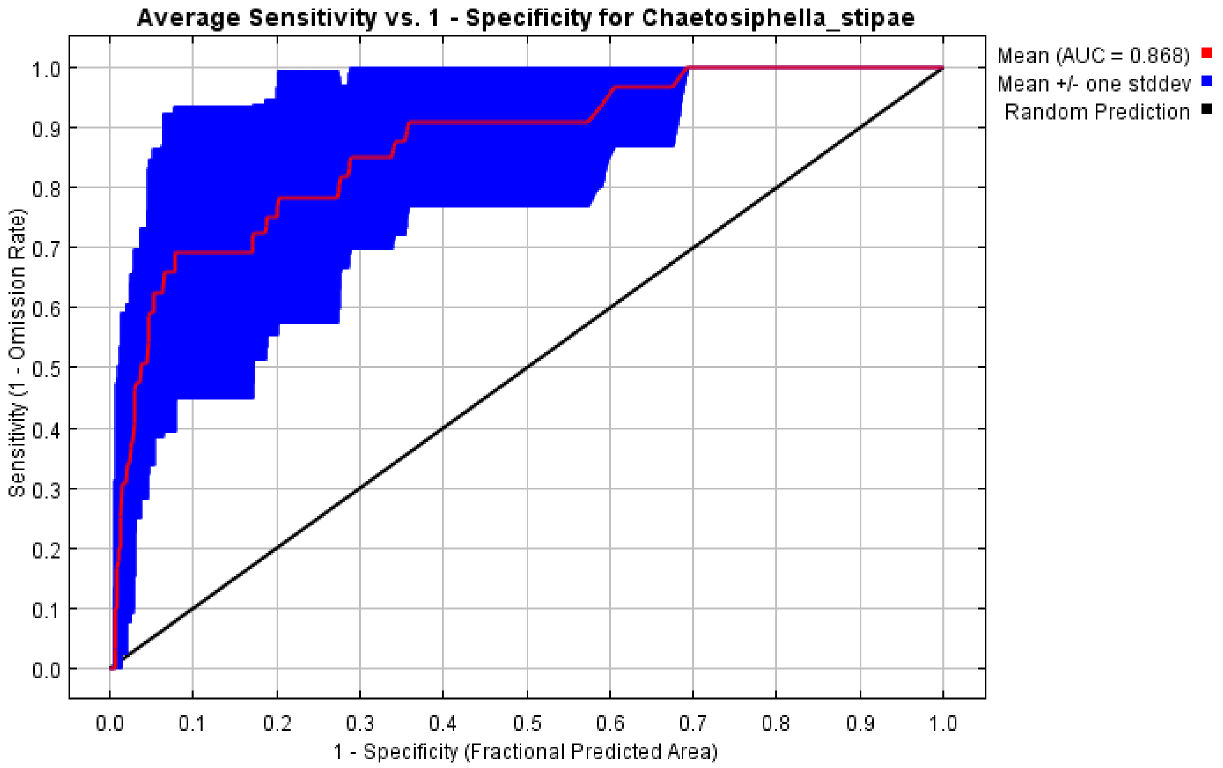


**Figure S1.2.** The receiver operating characteristic (ROC) curve generated in Maxent, showing an average of 10 repetitions of the model for *Ch. stipae stipae* (climate variables with output model for host plants).

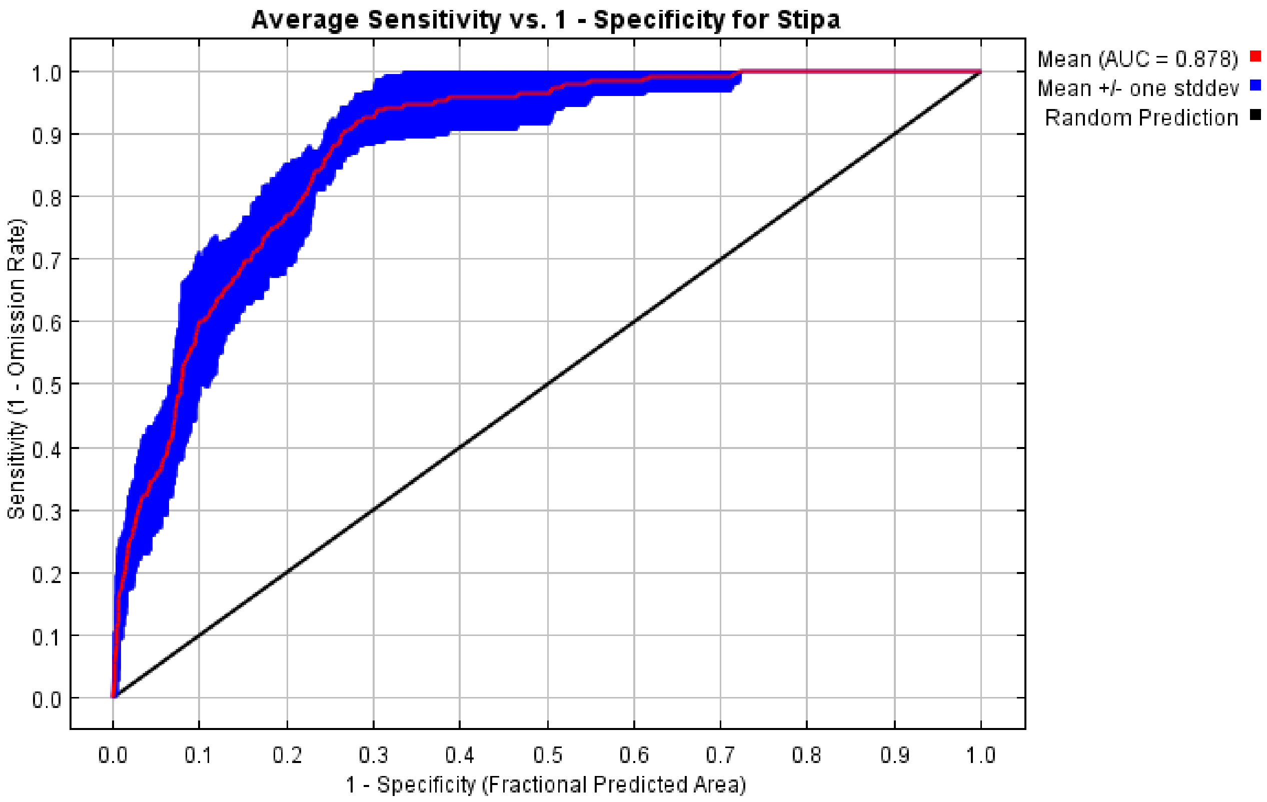


**Figure S1.3.** The receiver operating characteristic (ROC) curve generated in Maxent, showing an average of 10 repetitions of the model for representatives of the genus *Stipa*.

**(a) *Ch. stipae stipae* – only climate variables**

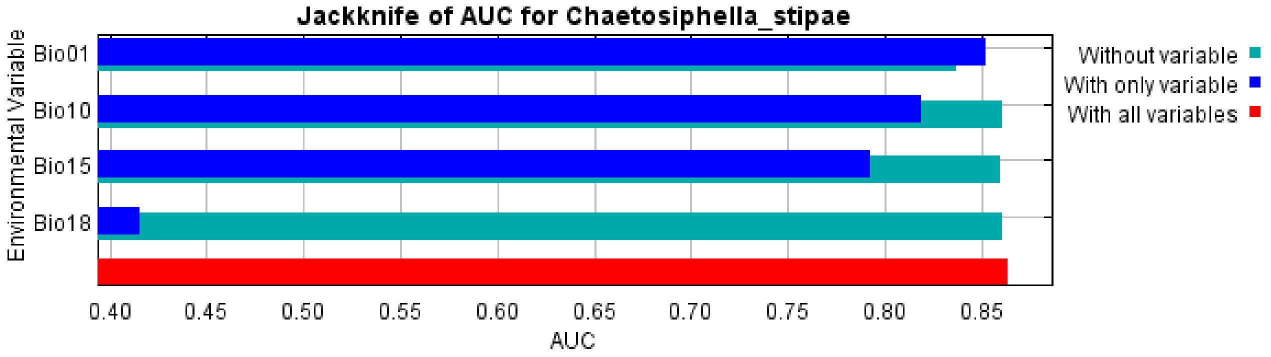


**(b) *Ch. stipae stipae* – climate variables with model for host plants**

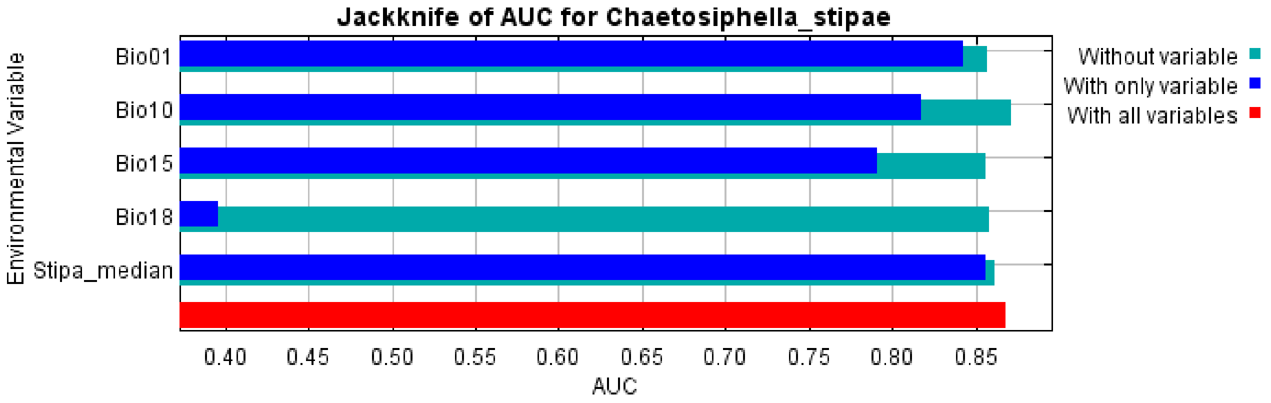


**(c) representatives of the genus *Stipa***

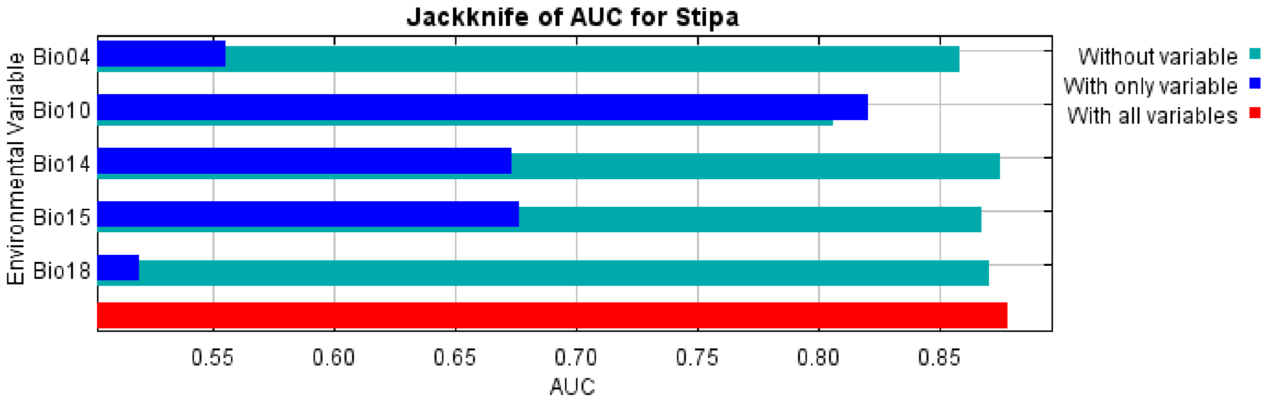


**Figure S1.4.** Results of jackknife test of variable importance using AUC on test data for (a) *Ch. stipae stipae* – only climate variables, (b) *Ch. stipae stipae* – climate variables with output model for host plants and (c) representatives of the genus *Stipa*. The jackknife test in blue bars shows individual environmental variable importance relative to the red bar which shows all environmental variables. Light blue bar shows whether a variable has any information that isn't present in the other variables, and a dark blue bar shows whether a variable has any useful information by itself. Values shown are averages over replicate runs.

**(a) *Ch. stipae stipae* – only climate variables**

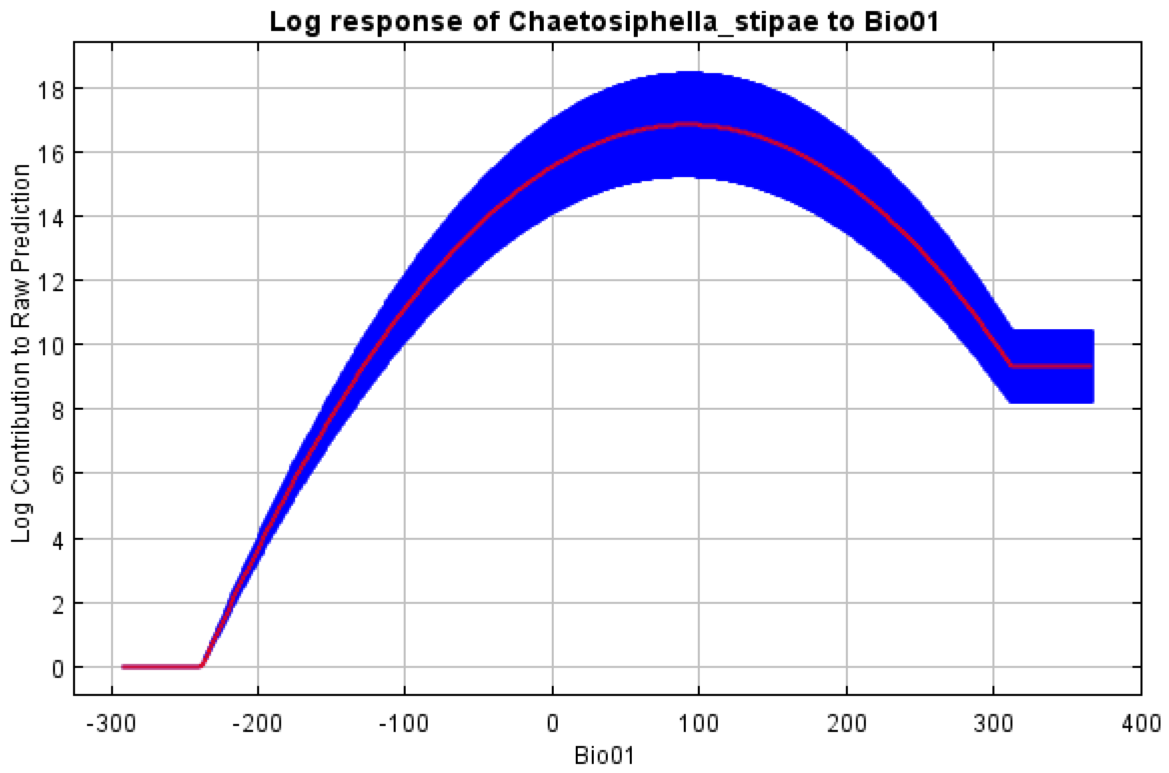


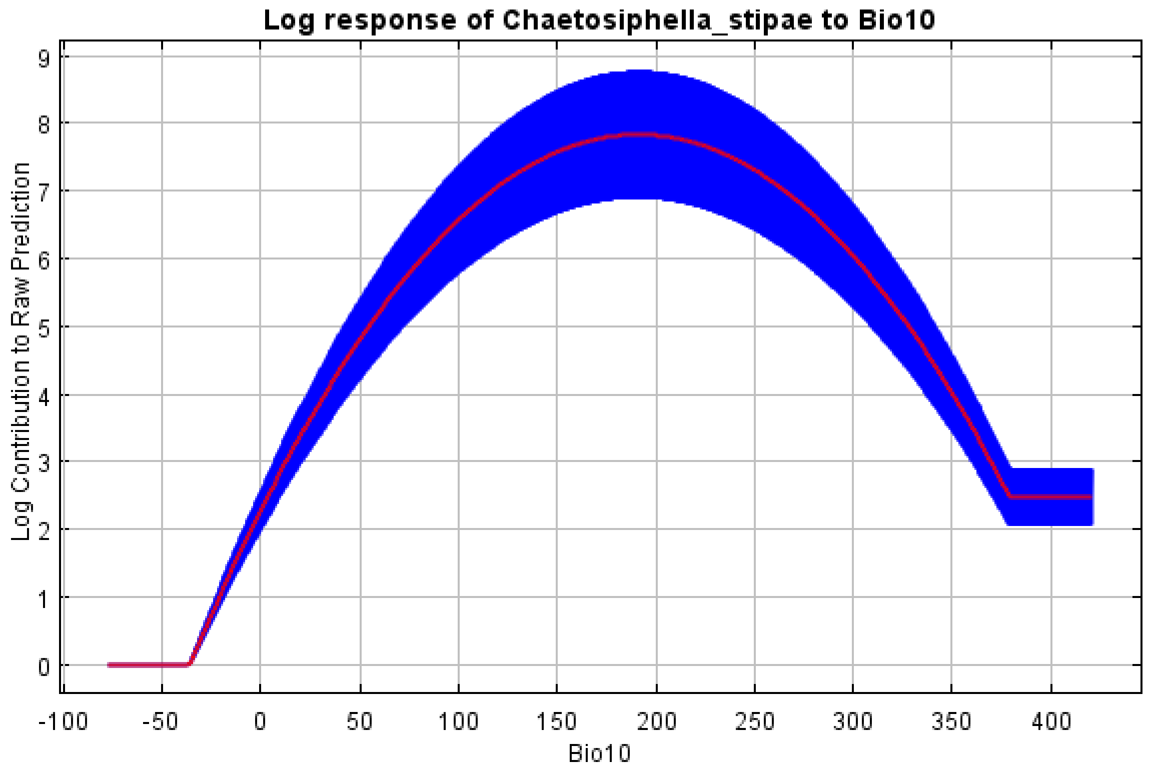

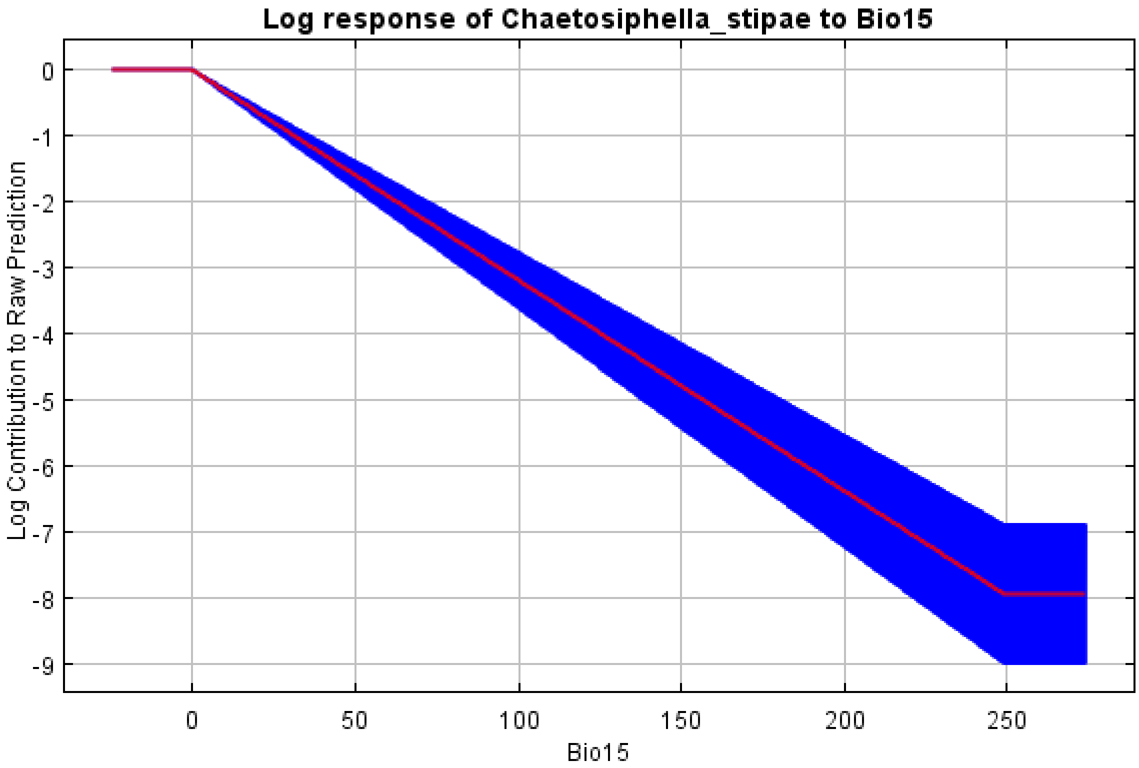
Log response of Chaetosiphella_stipae to Bio15
Log Contribution to Raw Prediction
0
-1
-2
-3
-4
-5
-6
-7
-8
-9
0
50
100
150
200
250
Bio15

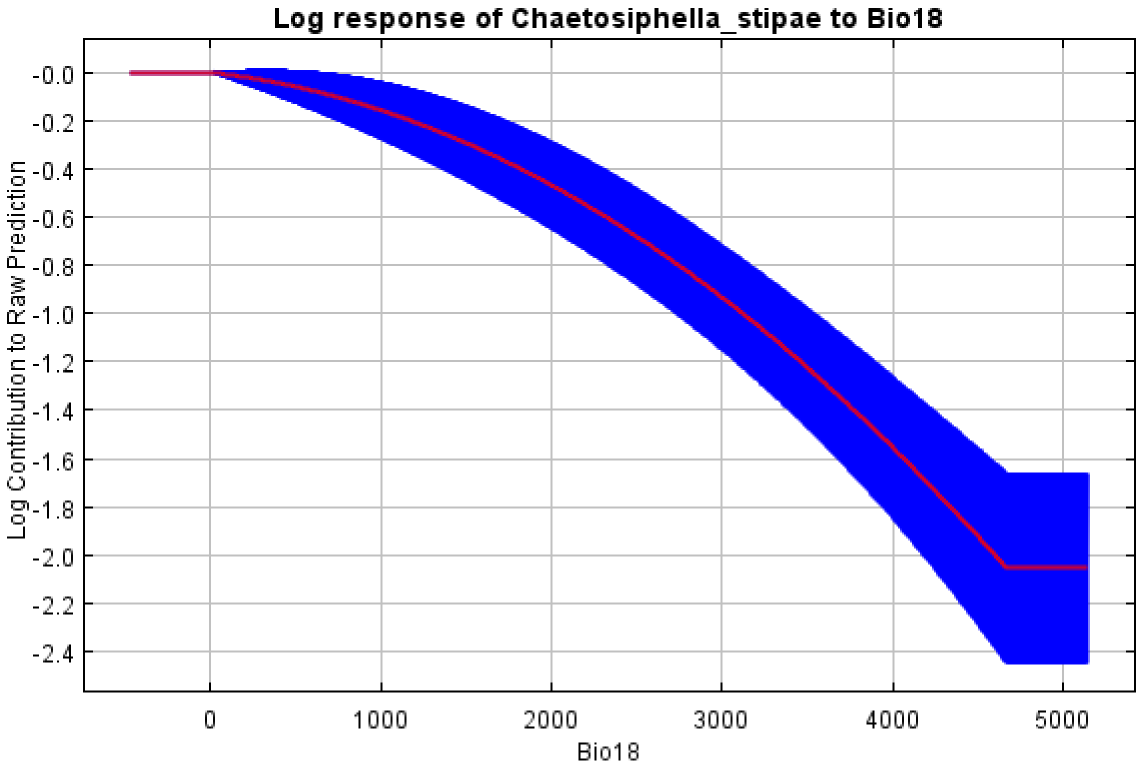
Log response of Chaetosiphella_stipae to Bio18
Log Contribution to Raw Prediction
-0.0
-0.2
-0.4
-0.6
-0.8
-1.0
-1.2
-1.4
-1.6
-1.8
-2.0
-2.2
-2.4
0
1000
2000
3000
4000
5000
Bio18

**(b) *Ch. stipae stipae* – climate variables with model for host plants**

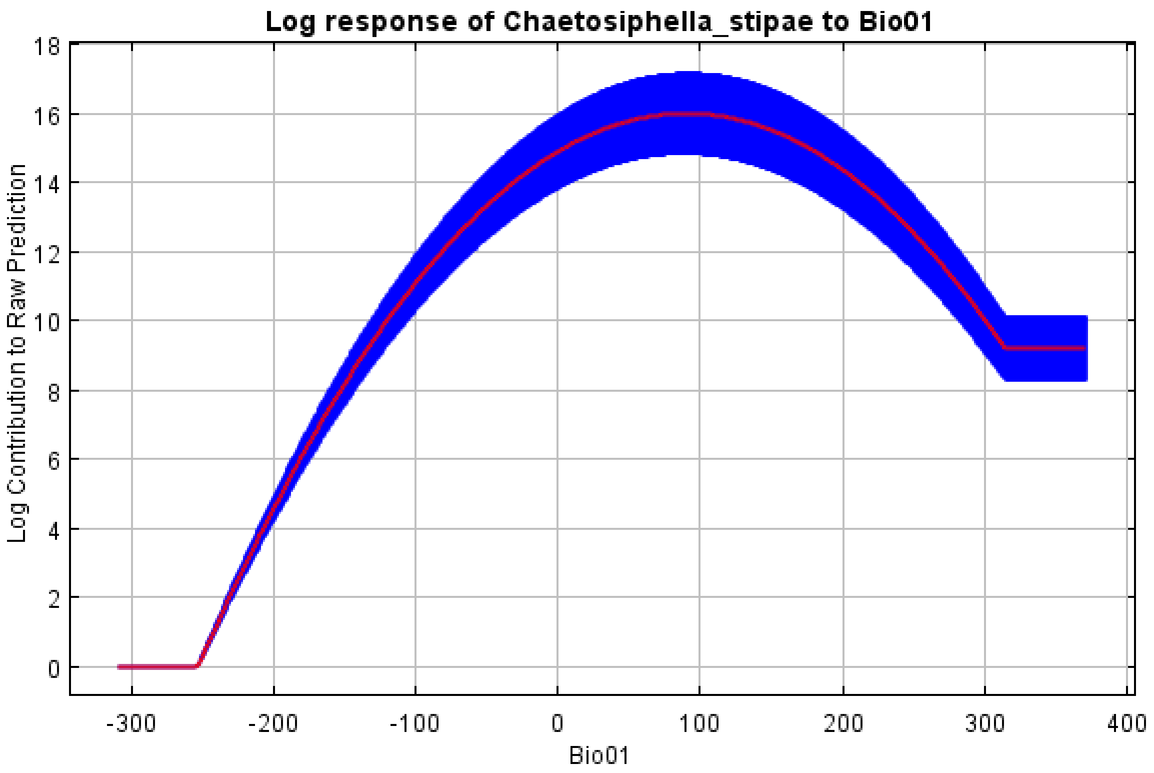


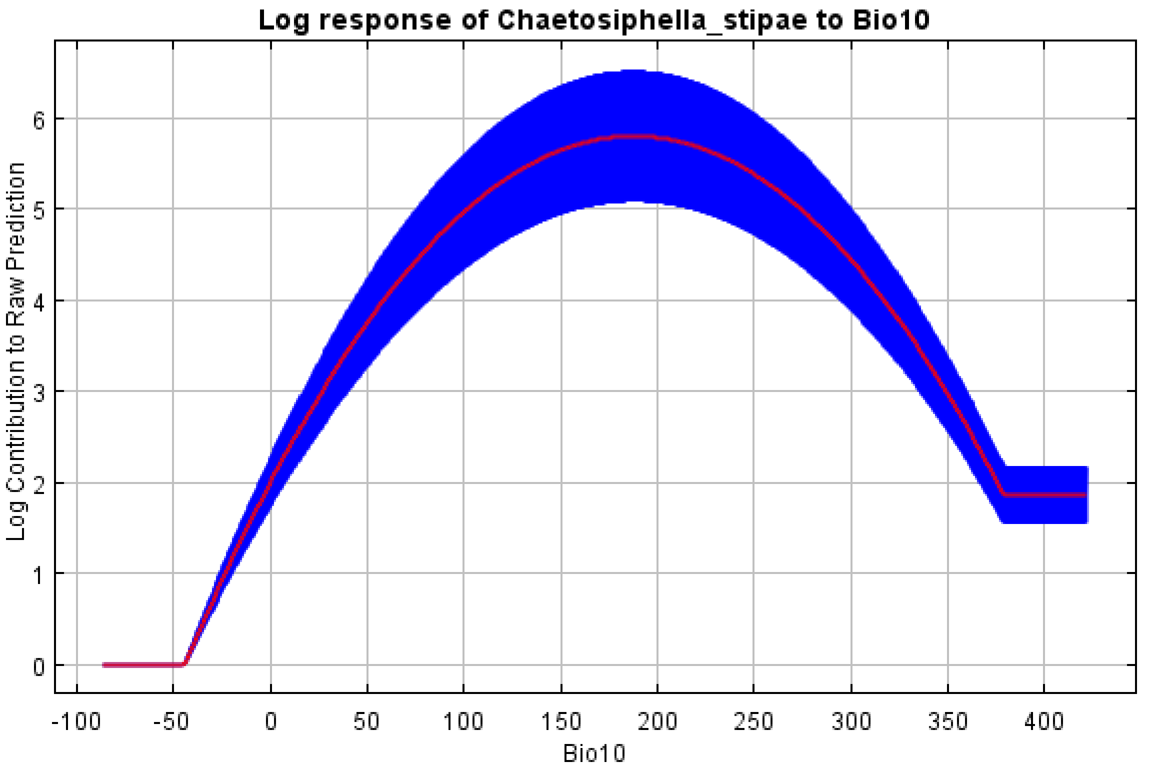

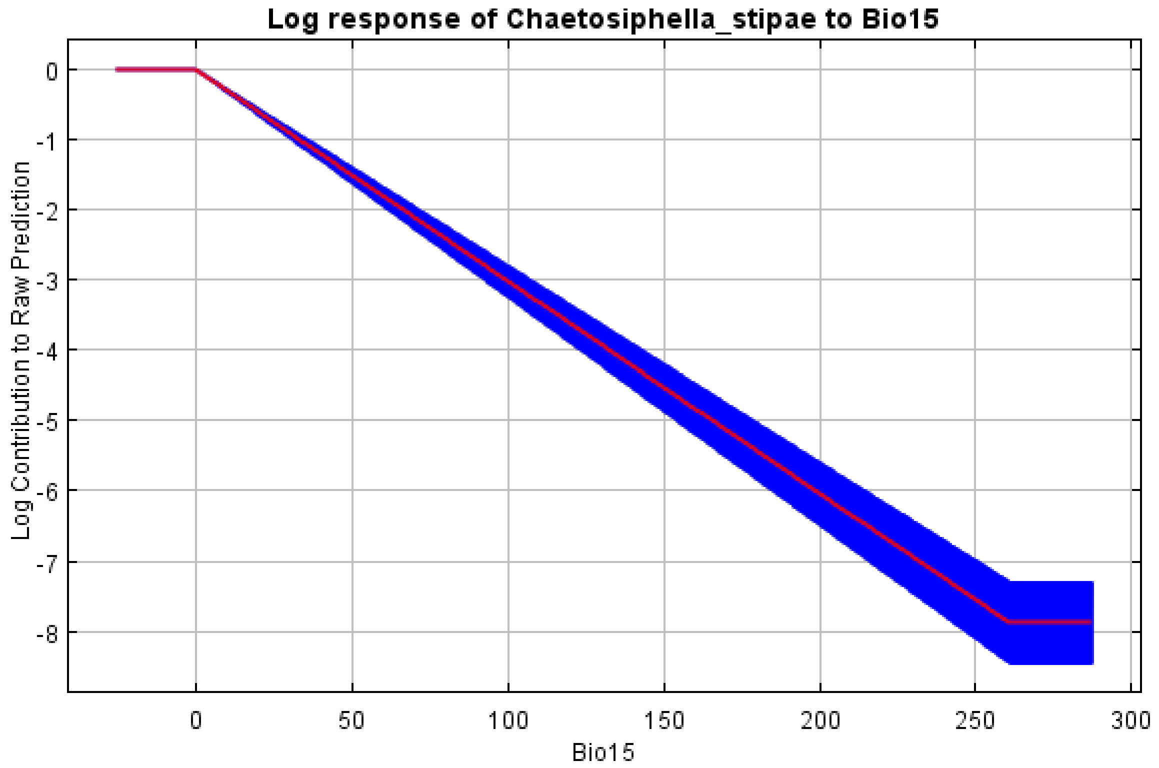
Log response of Chaetosiphella_stipae to Bio15
Log Contribution to Raw Prediction
0
-1
-2
-3
-4
-5
-6
-7
-8
0
50
100
150
200
250
300
Bio15

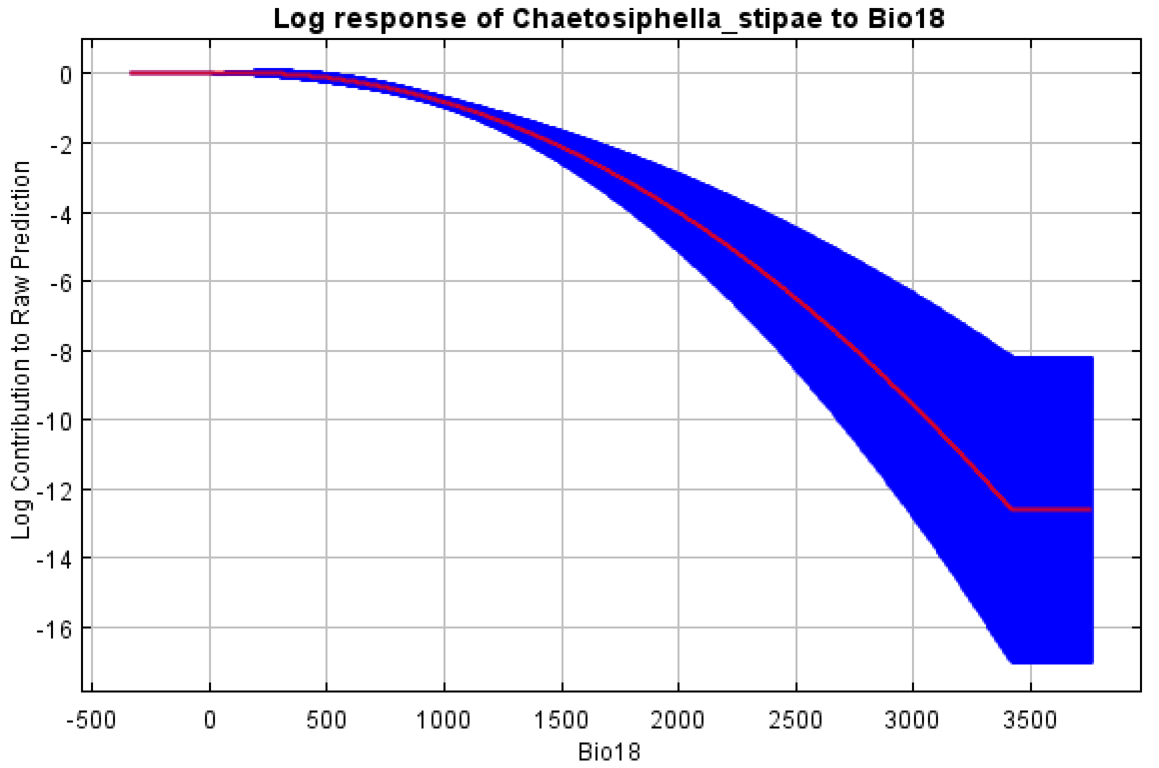
Log response of Chaetosiphella_stipae to Bio18
Log Contribution to Raw Prediction
0
-2
-4
-6
-8
-10
-12
-14
-16
-500
0
500
1000
1500
2000
2500
3000
3500
Bio18

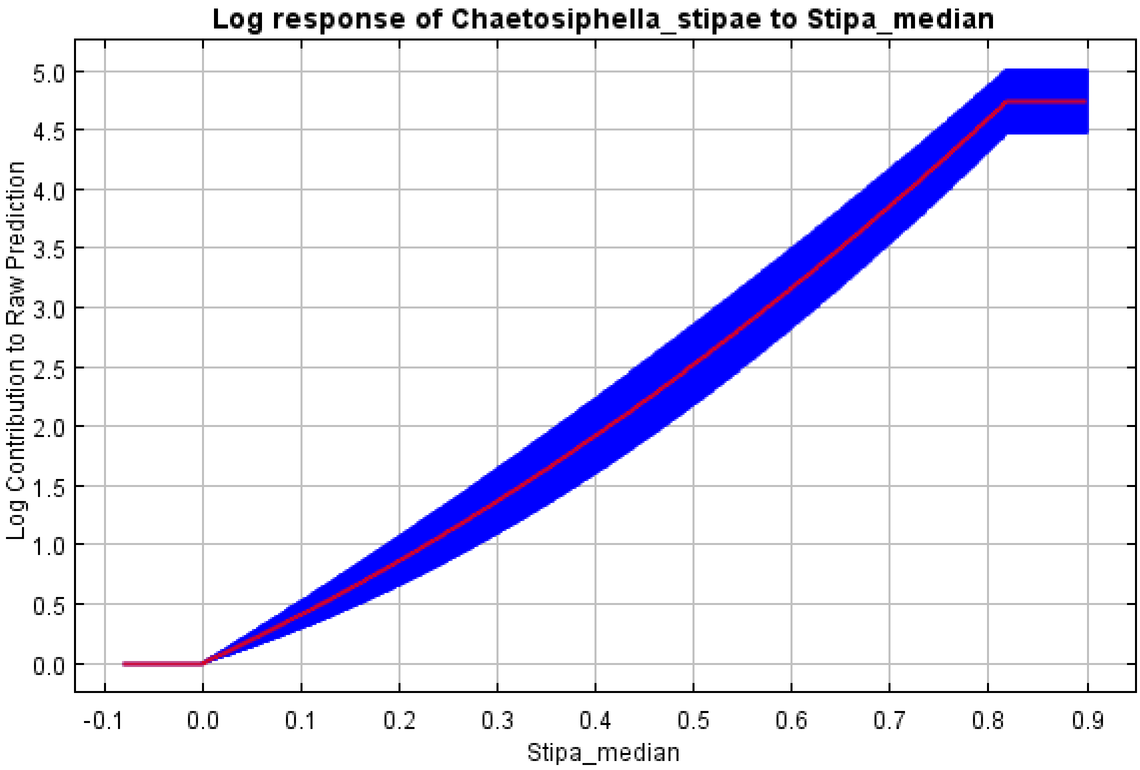


**(c) representatives of the genus *Stipa***

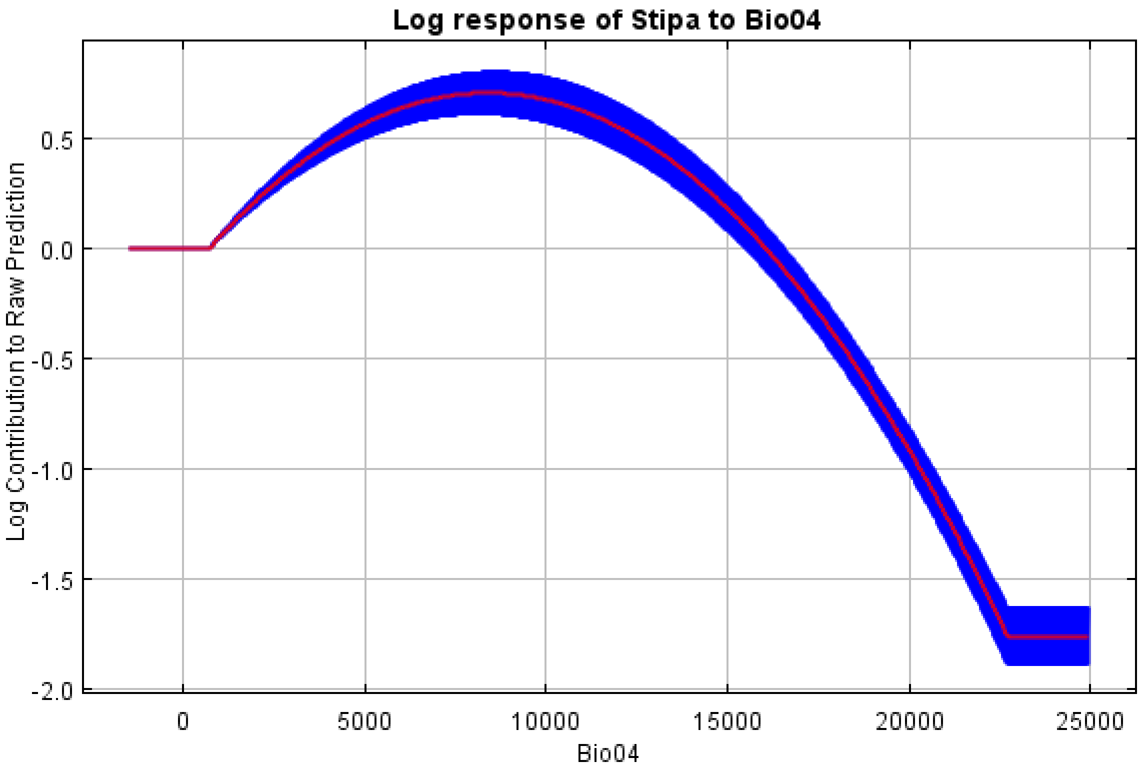

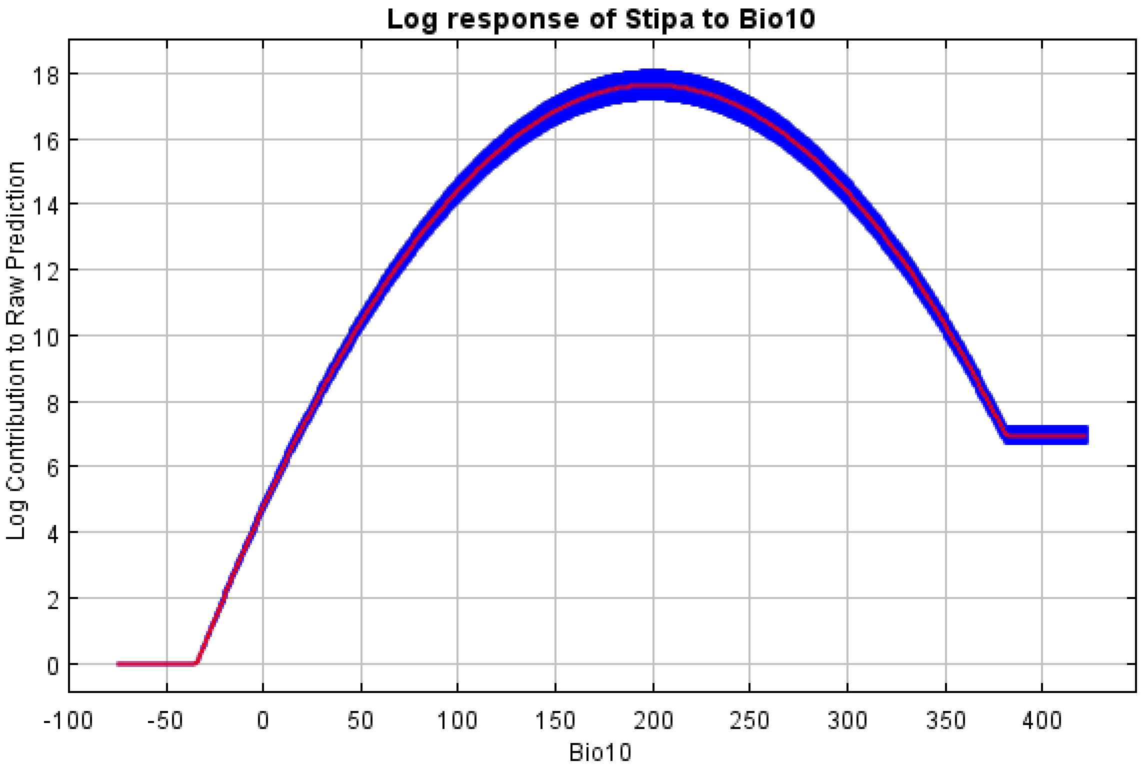

Log response of Stipa to Bio10
Log Contribution to Raw Prediction
Bio10
18
16
14
12
10
8
6
4
2
0
-100
-50
0
50
100
150
200
250
300
350
400


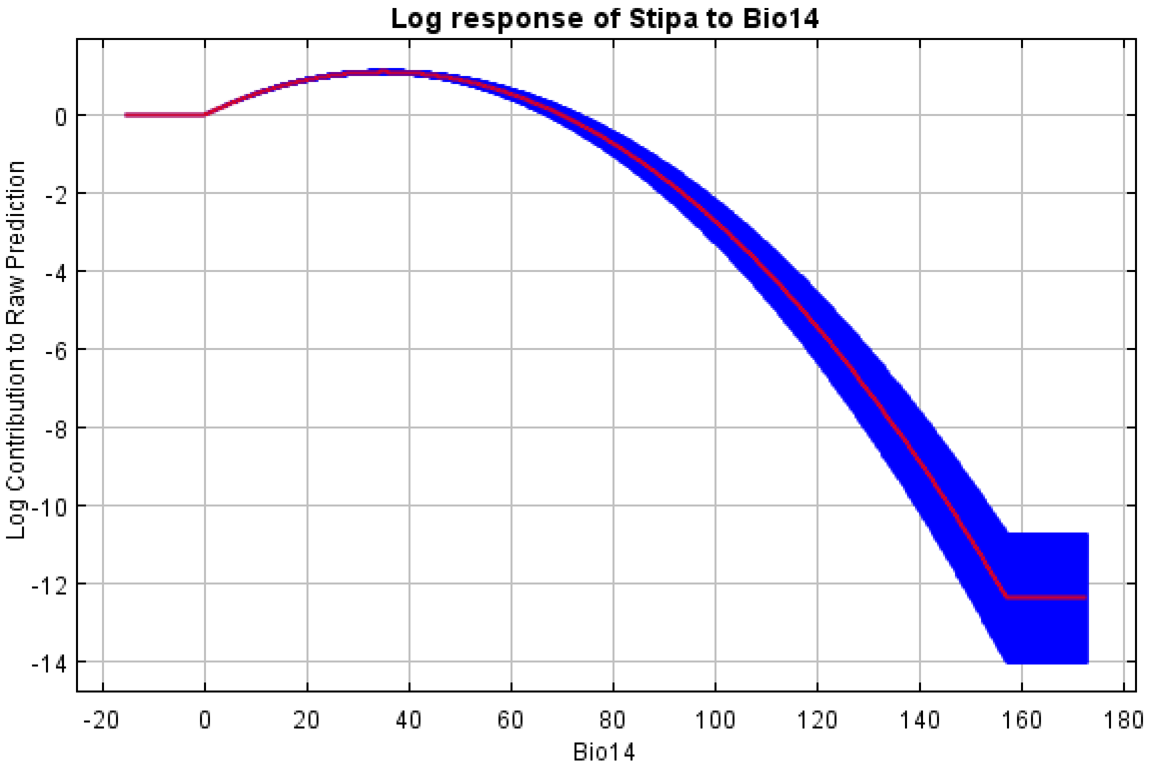

Log response of Stipa to Bio14
Log Contribution to Raw Prediction
Bio14
0
-2
-4
-6
-8
-10
-12
-14
-20
0
20
40
60
80
100
120
140
160
180

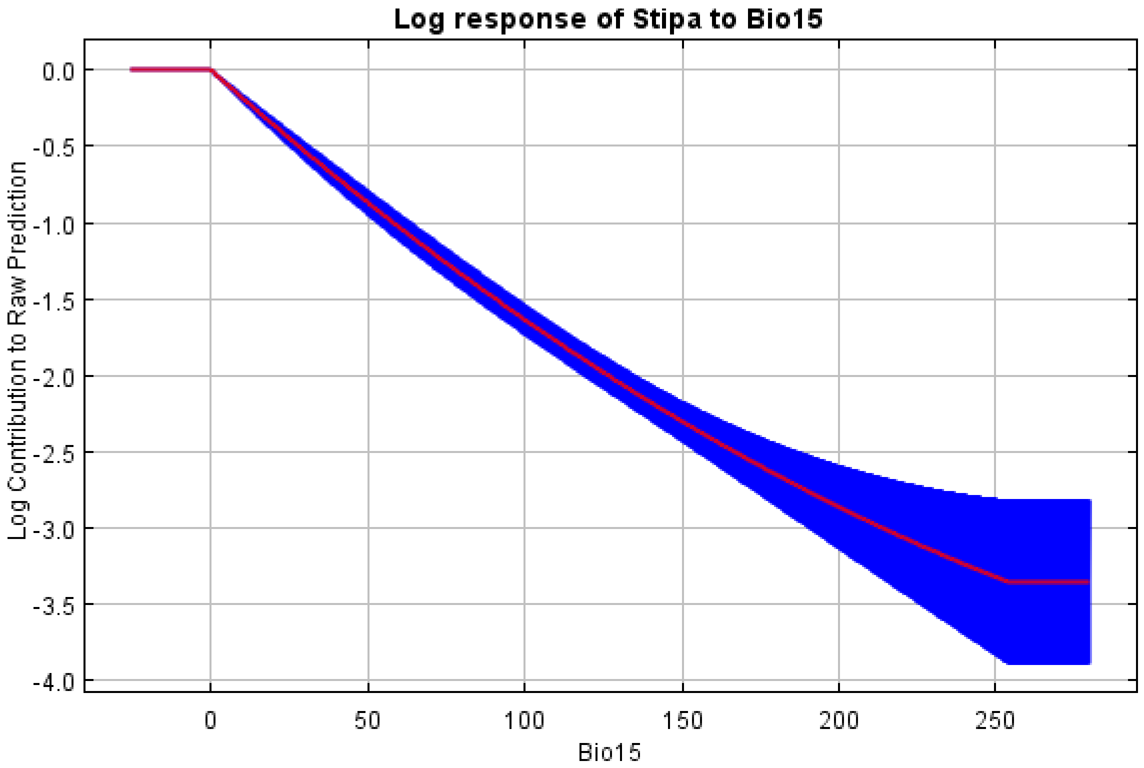


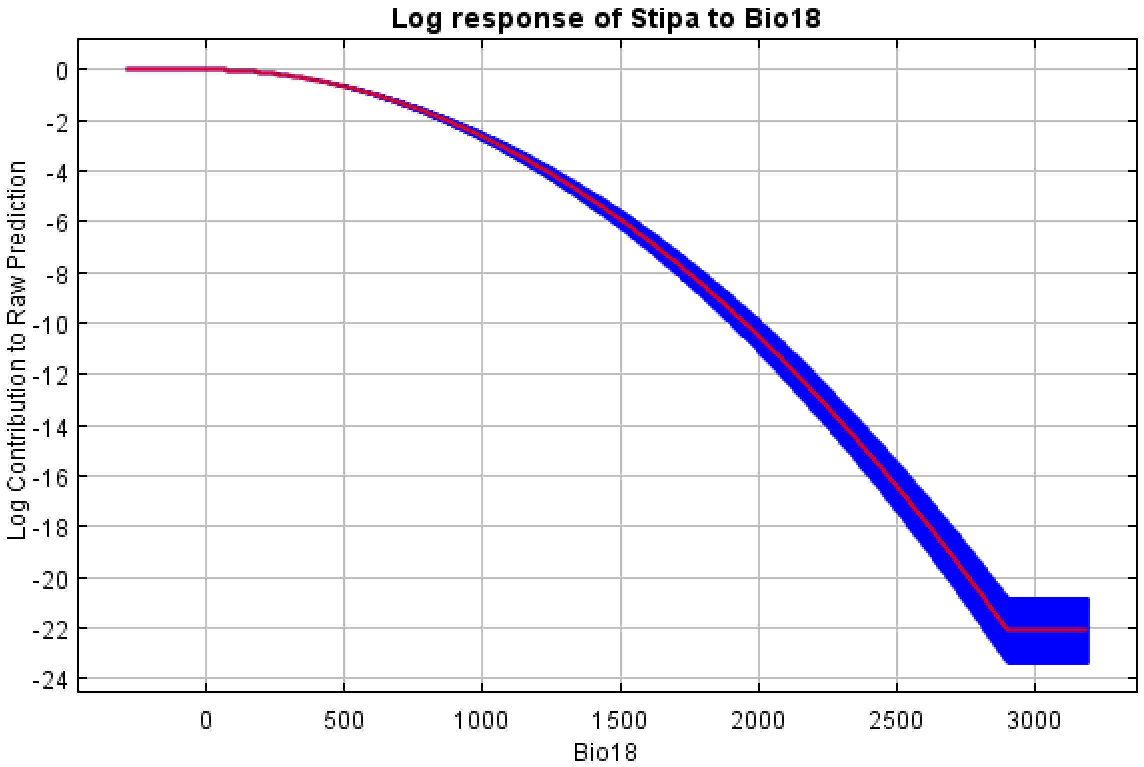


**Figure S1.5.** Results of response curves for a selected variables for (a) *Ch. stipae stipae* – only climate variables, (b) *Ch. stipae stipae* – climate variables with output model for host plants and (c) representatives of the genus *Stipa*. The curves show the mean response of the 10 replicate Maxent runs (red) and the mean +/- one standard deviation (blue, two shades for categorical variables).

(a)

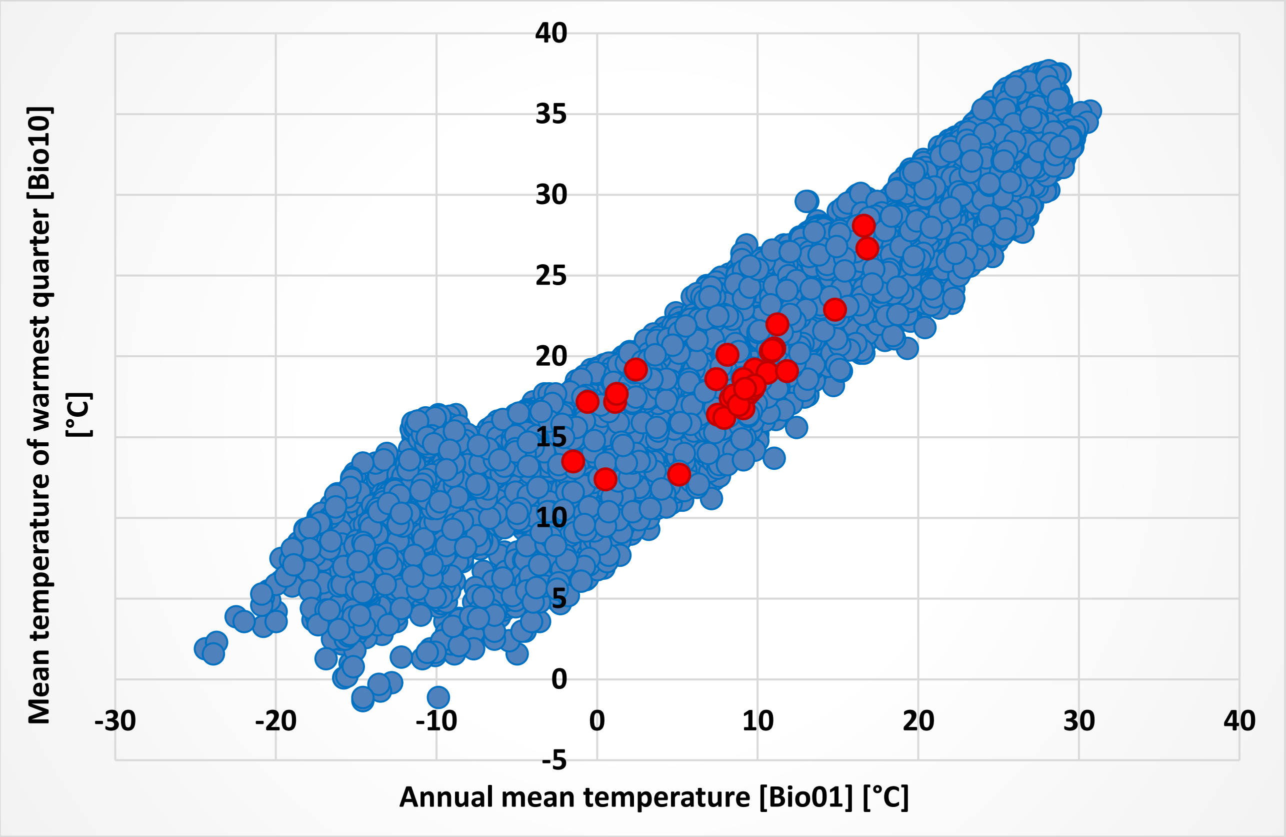

Mean temperature of warmest quarter [Bio10] [°C]
40
35
30
25
20
15
10
5
0
-5
-30
-20
-10
0
10
20
30
40
Annual mean temperature [Bio01] [°C]


(b)

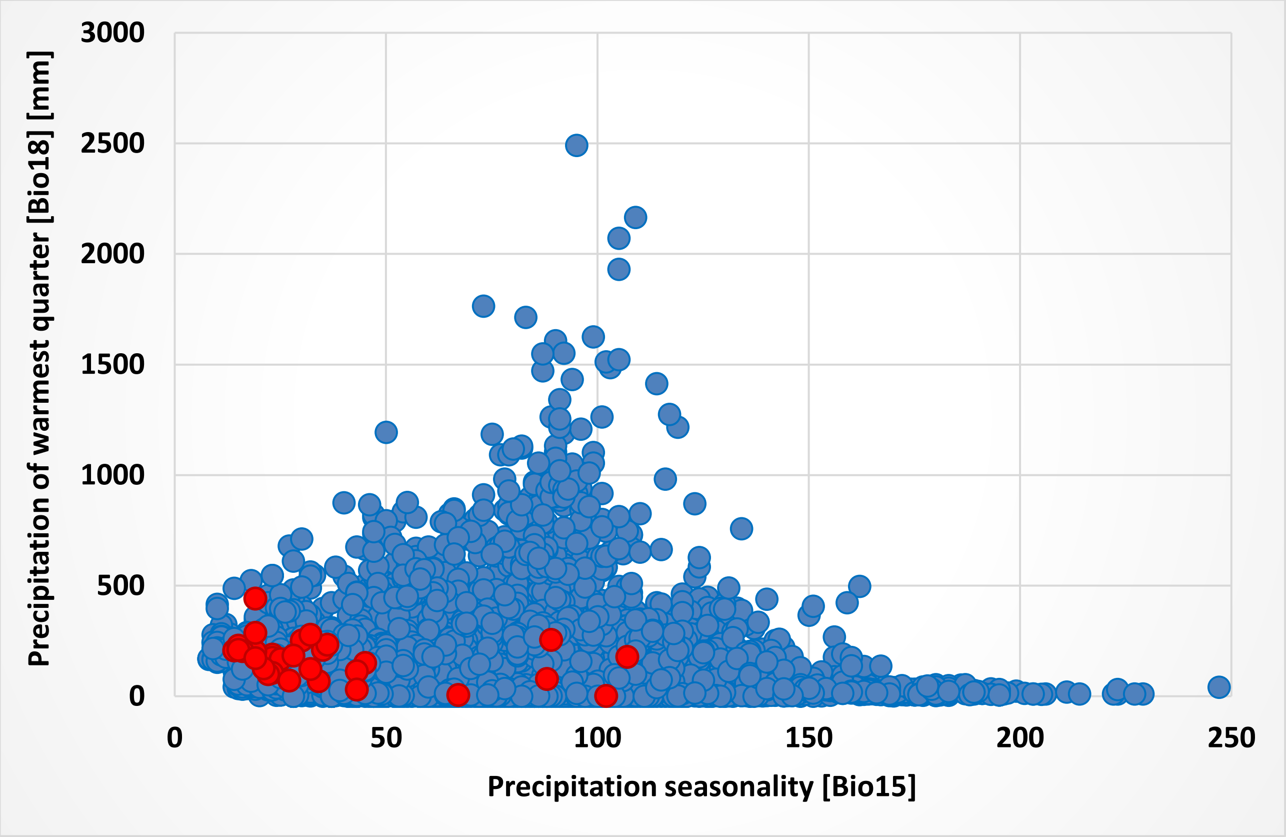

Precipitation of warmest quarter [Bio18] [mm]
3000
2500
2000
1500
1000
500
0
0
50
100
150
200
250
Precipitation seasonality [Bio15]

**(c)**

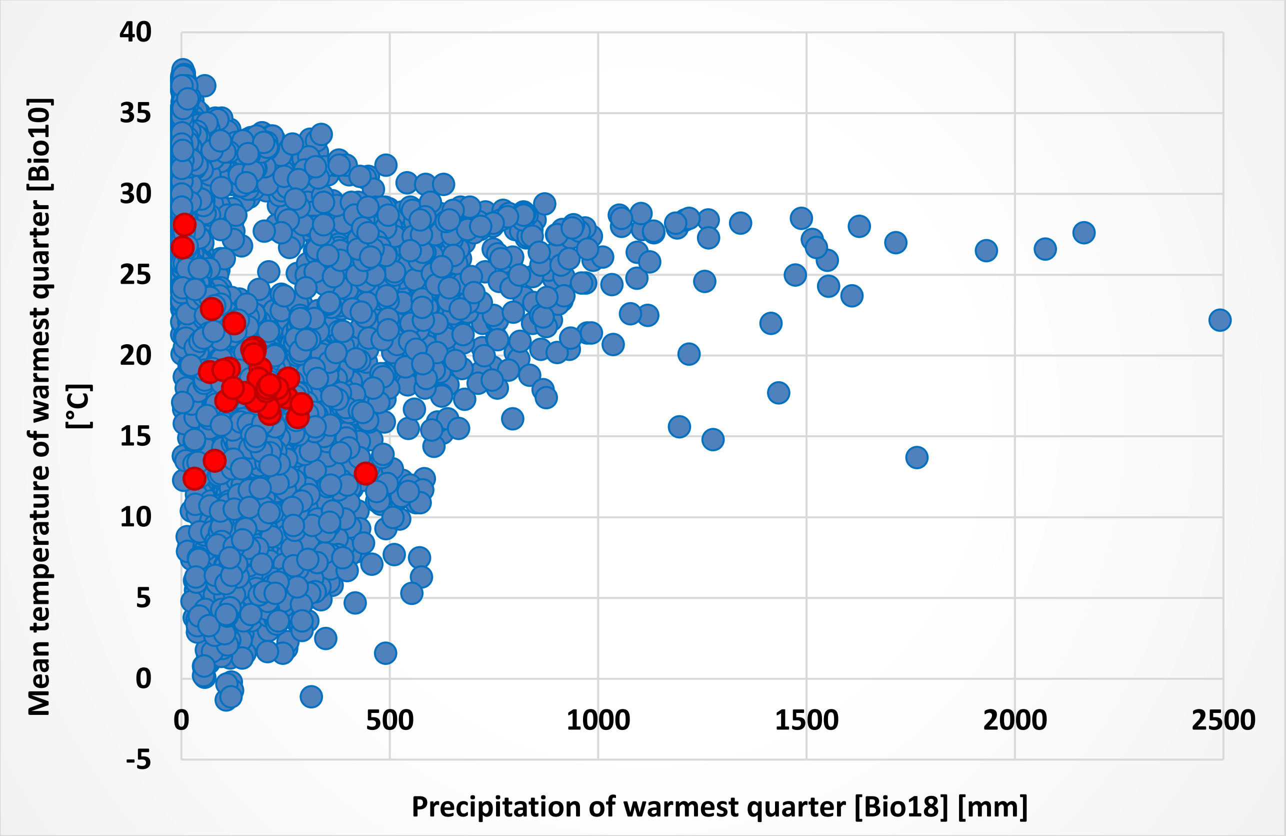


**Figure S1.6.** The locations of *Chaetosiphella stipae stipae* records in the multidimensional climatic space (red circles). (a) climatic space defined by annual mean temperature and mean temperature of warmest quarter; (b) climatic space defined by precipitation seasonality and precipitation of warmest quarter; (c) climatic space defined by mean temperature of warmest quarter and precipitation of warmest quarter. Blue dots: climatic space defined by 10000 background points.

(a)

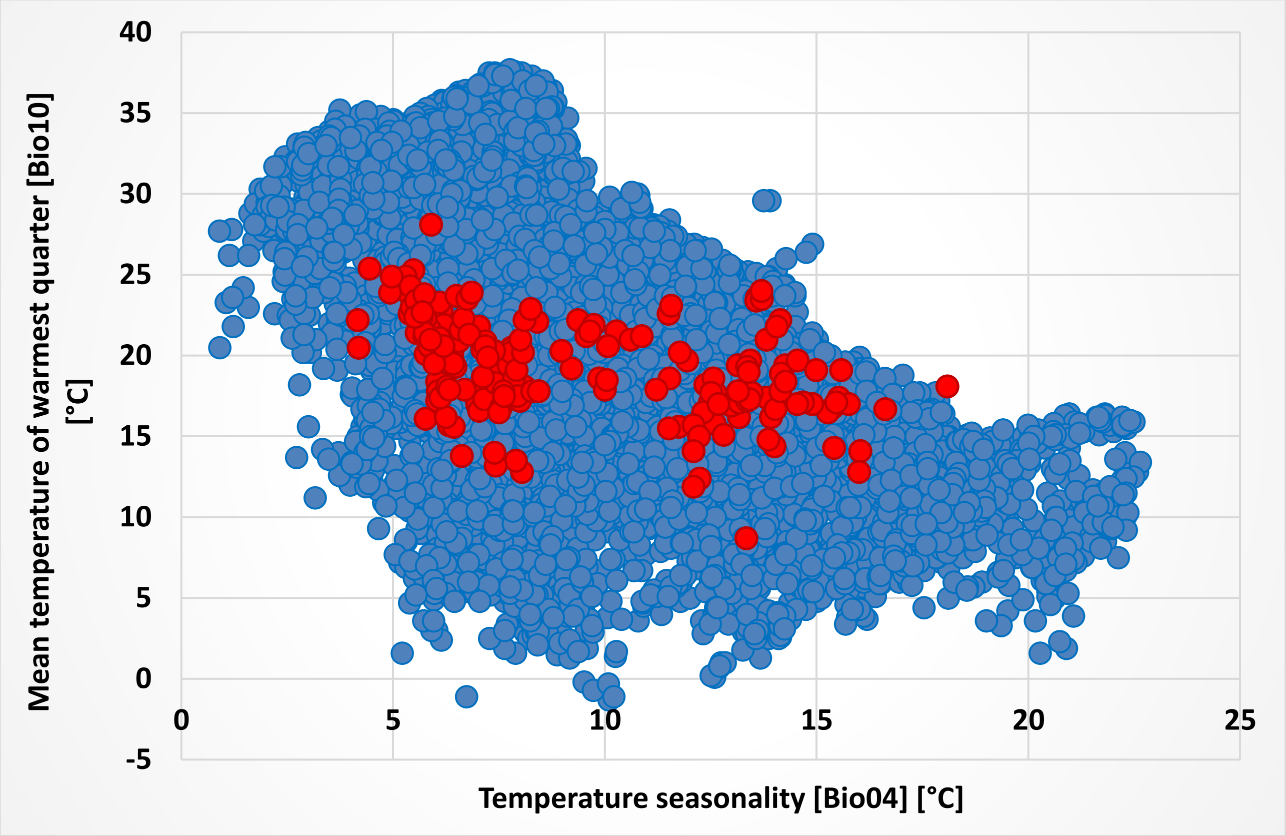

Mean temperature of warmest quarter [Bio10] [°C]
40
35
30
25
20
15
10
5
0
-5
0
5
10
15
20
25
Temperature seasonality [Bio04] [°C]


(b)

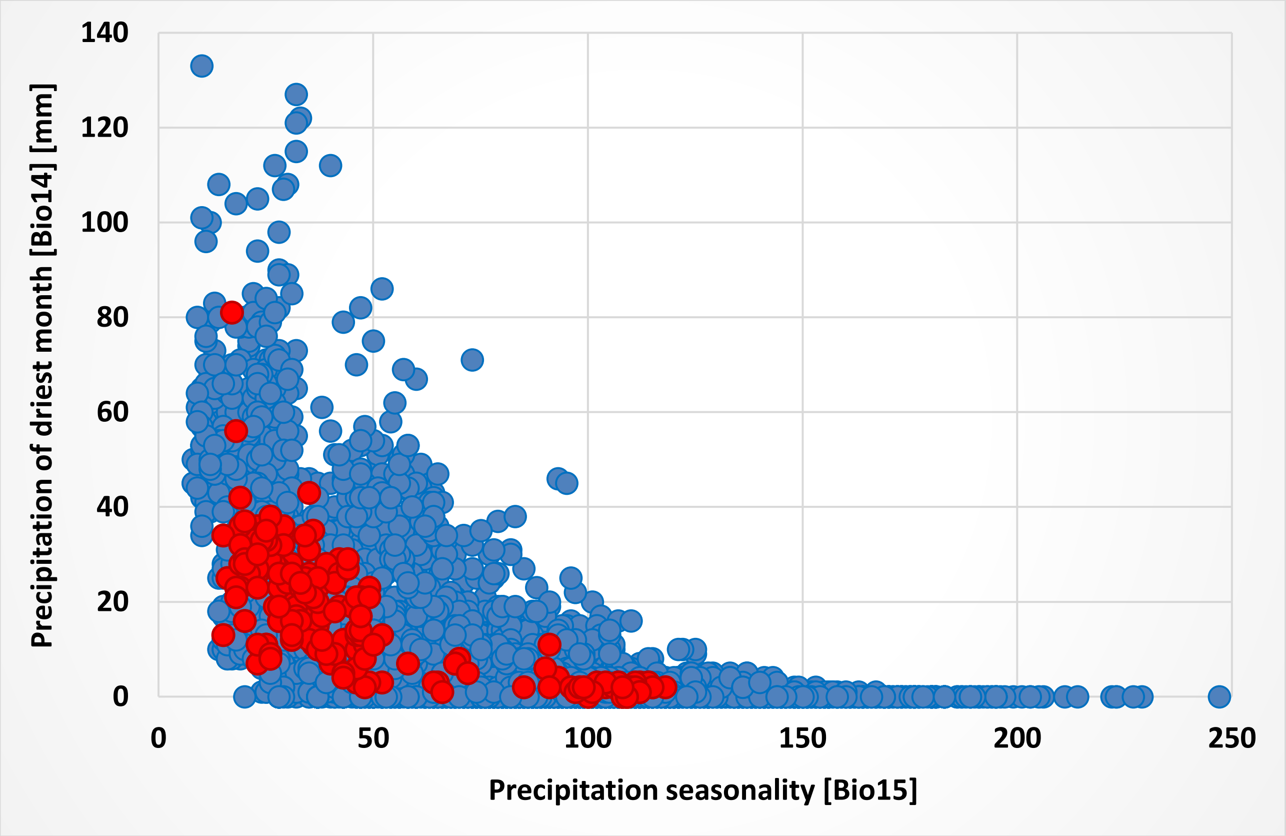

Precipitation of driest month [Bio14] [mm]
140
120
100
80
60
40
20
0
0
50
100
150
200
250
Precipitation seasonality [Bio15]

(c)

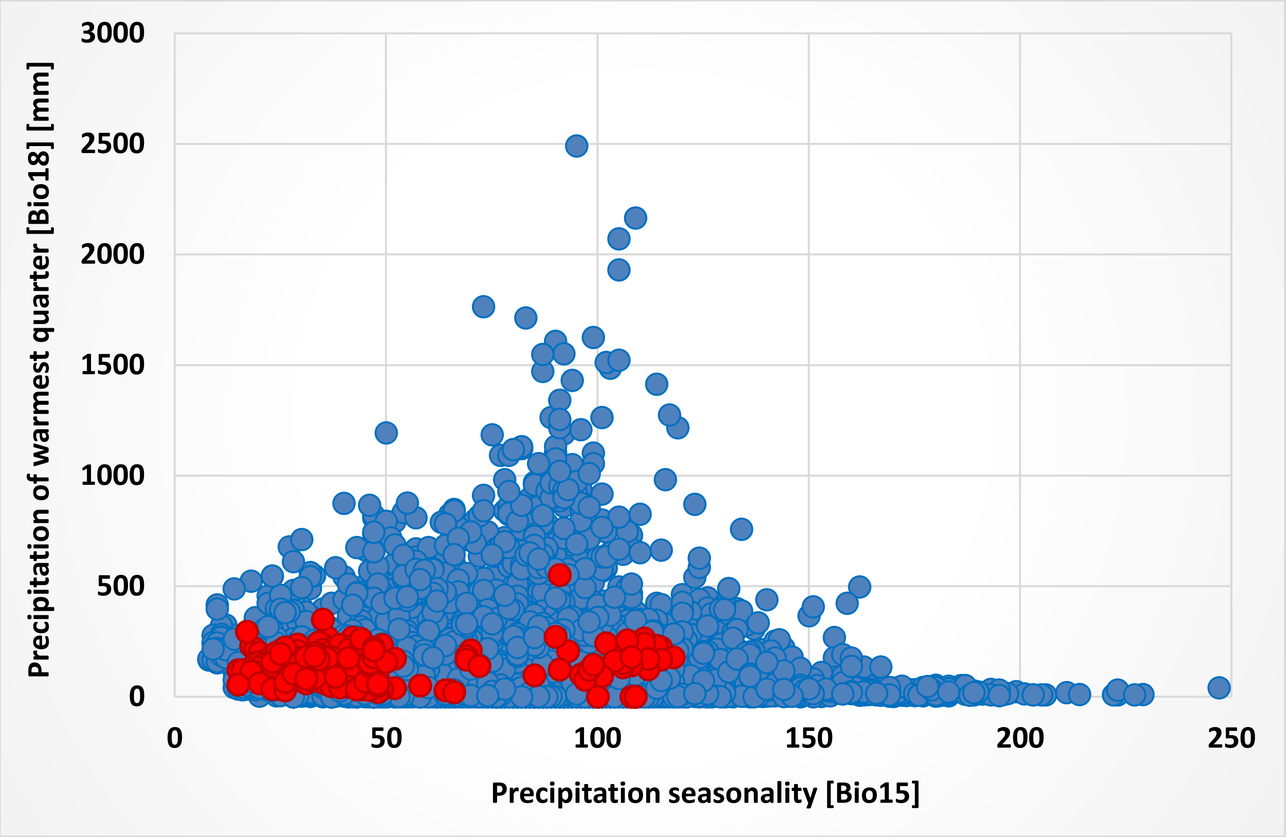

Precipitation of warmest quarter [Bio18] [mm]
3000
2500
2000
1500
1000
500
0
0
50
100
150
200
250
Precipitation seasonality [Bio15]


(d)

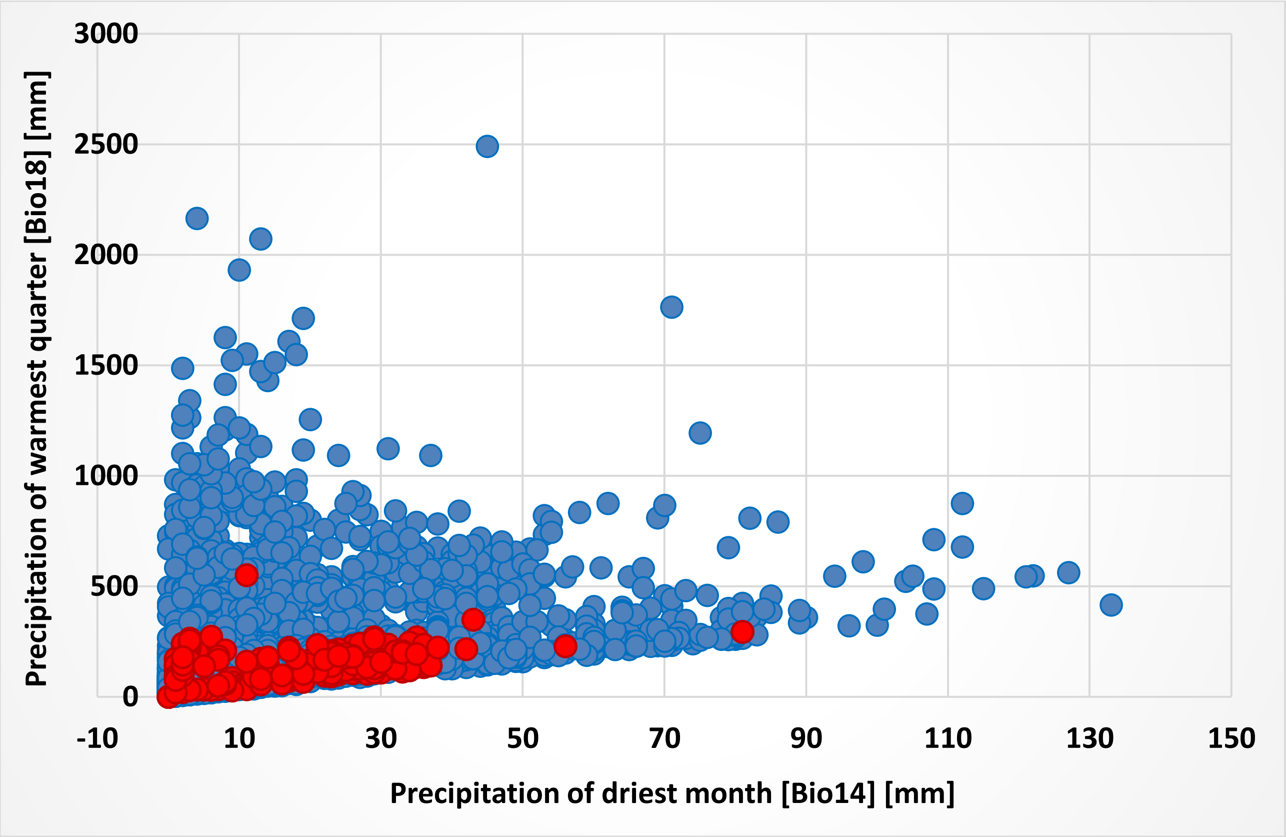

Precipitation of warmest quarter [Bio18] [mm]
3000
2500
2000
1500
1000
500
0
-10
10
30
50
70
90
110
130
150
Precipitation of driest month [Bio14] [mm]

**(e)**

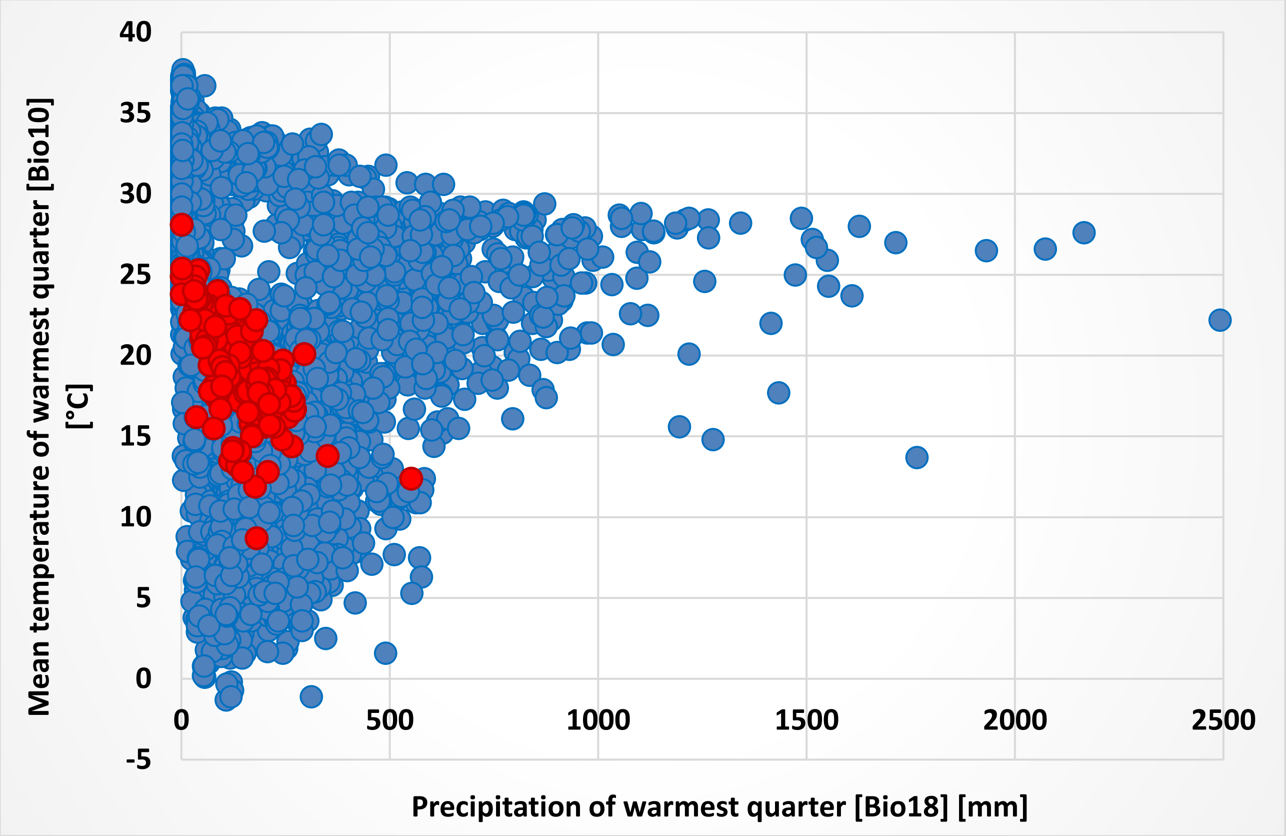


**Figure S1.7.** The locations of *Stipa* species records in the multidimensional climatic space (red circles). (a) climatic space defined by temperature seasonality and mean temperature of warmest quarter; (b) climatic space defined by precipitation seasonality and precipitation of driest month; (c) climatic space defined by precipitation seasonality and precipitation of warmest quarter; (d) climatic space defined by precipitation of driest month and precipitation of warmest quarter; (e) climatic space defined by mean temperature of warmest quarter and precipitation of warmest quarter. Blue dots: climatic space defined by 10000 background points.

(a)

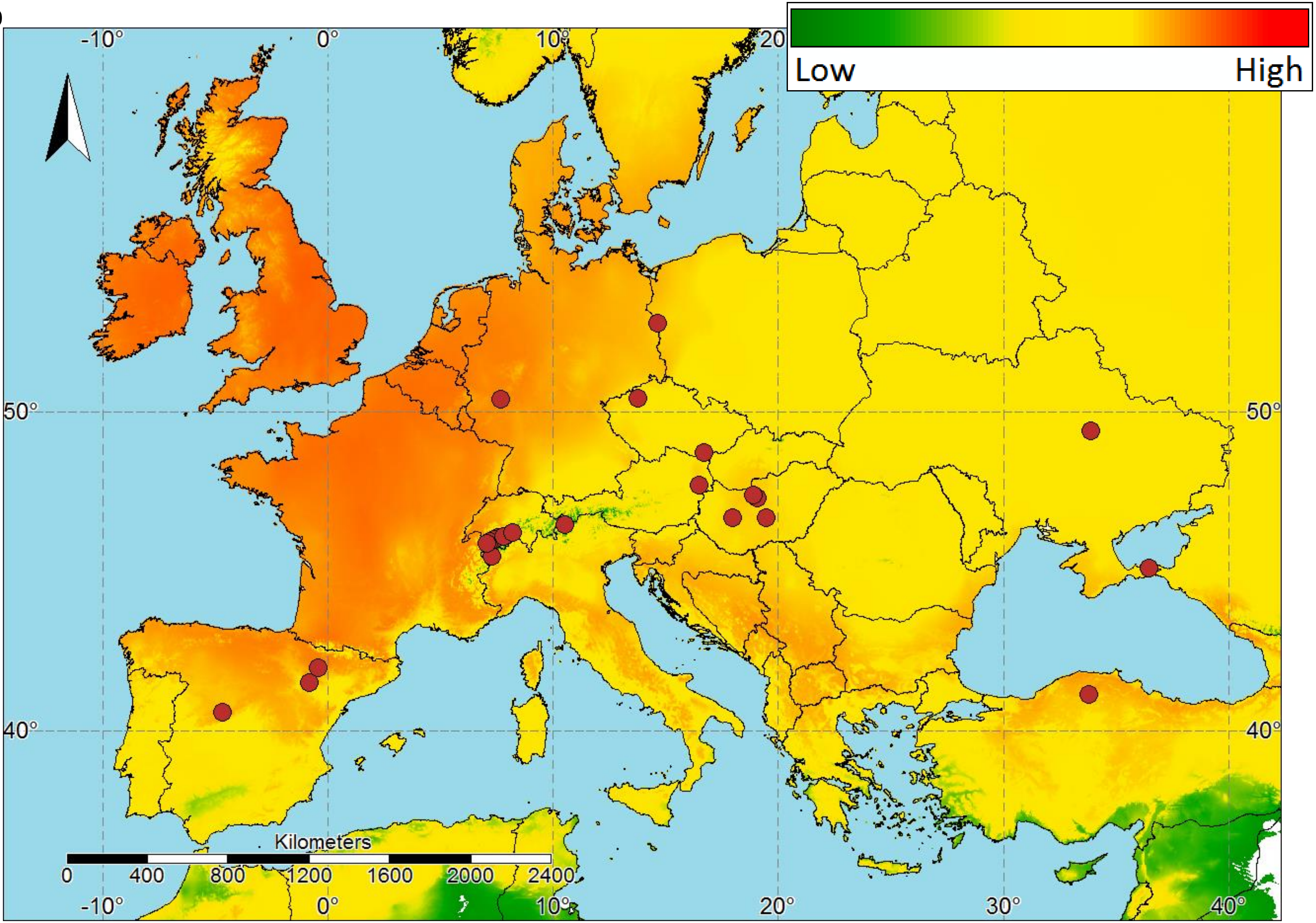
Low
High
-10°
0°
10°
20
20°
30°
40°
50°
40°
Kilometers
0
400
800
1200
1600
2000
2400

**(b)**

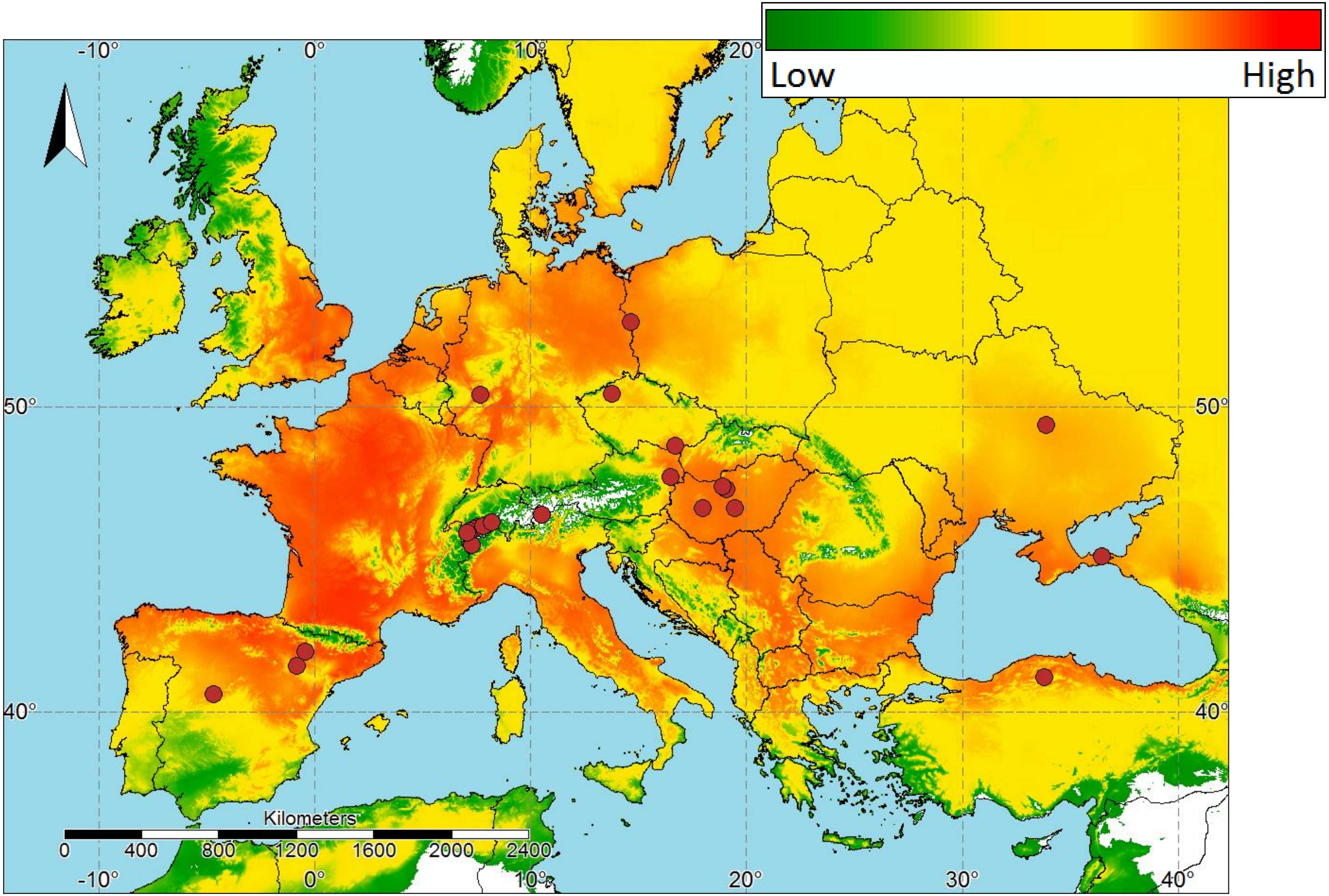


**Figure S1.8.** Detailed maps of potentially useful niche for *Chaetosiphella stipae stipae* in Europe with its known distribution. (a) Model based only on climate variables, and (b) model based on climate variables and outcome model of potential distribution of *Stipa* species. Maps were plotted using SAGA GIS 3.0.0[1] (http://www.saga-gis.org); projection – World Mercator (EPSG: 3395).

(a)

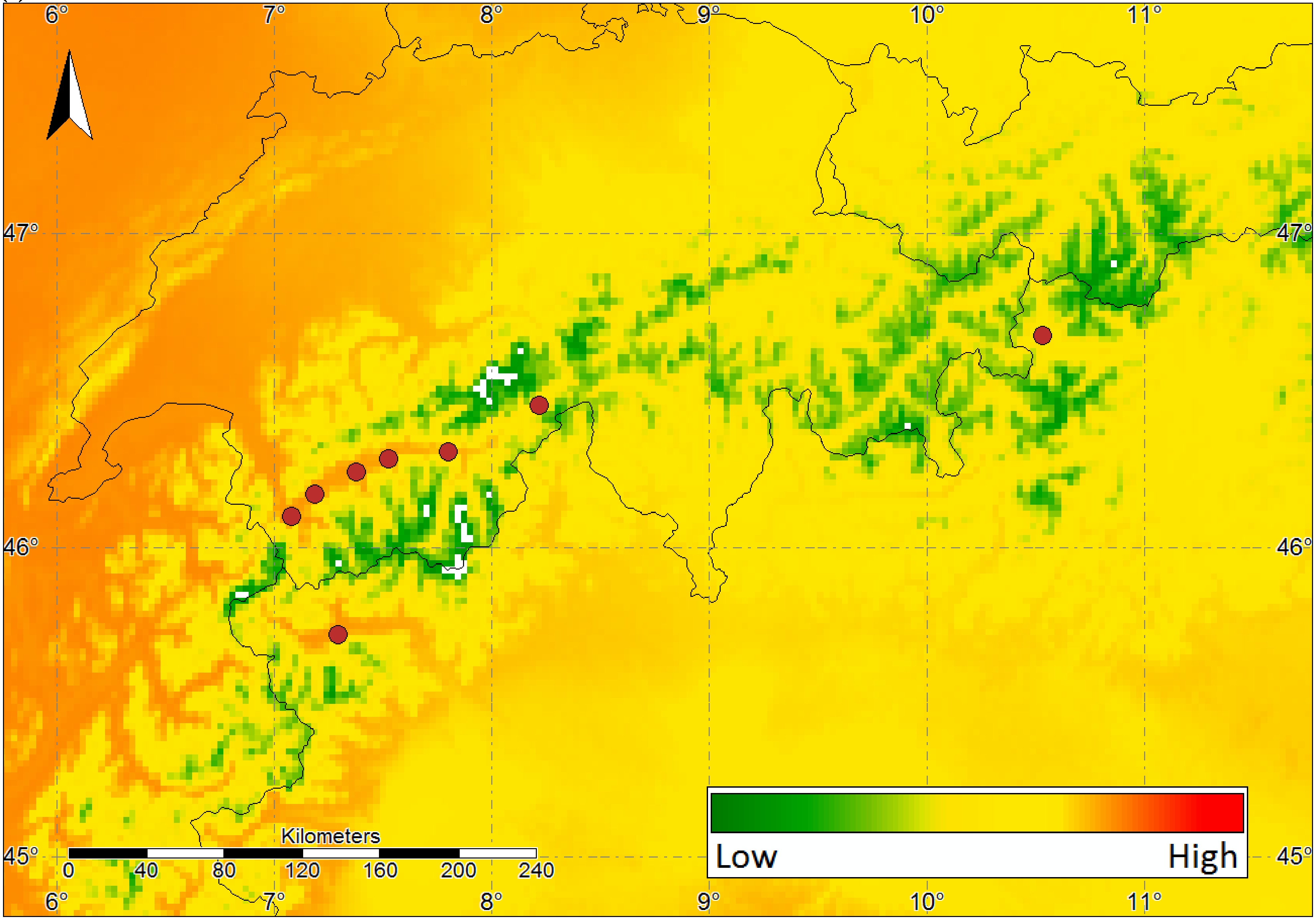

**(b)**

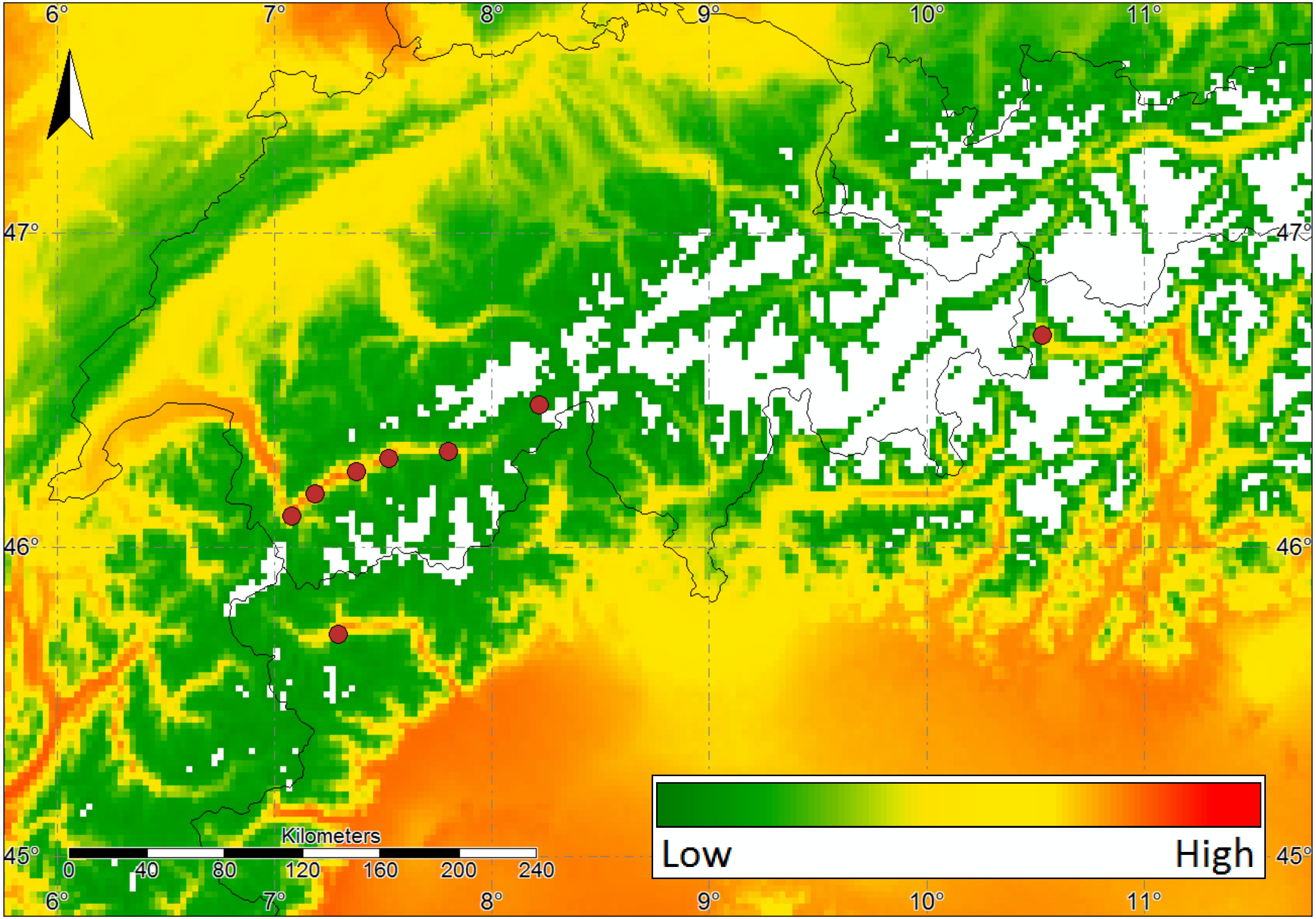


**Figure S1.9.** Potentially useful niche for *Ch. stipae stipae* in the Alps. (a) Model based only on climate variables, and (b) model based on climate variables and outcome model of potential distribution of *Stipa* species. Close-up map shows how the biotic layer affects mountain areas by significantly reducing the potentially suitable area for aphid. Maps were plotted using SAGA GIS 3.0.0[1] (http://www.saga-gis.org); projection – World Mercator (EPSG: 3395).

(a)

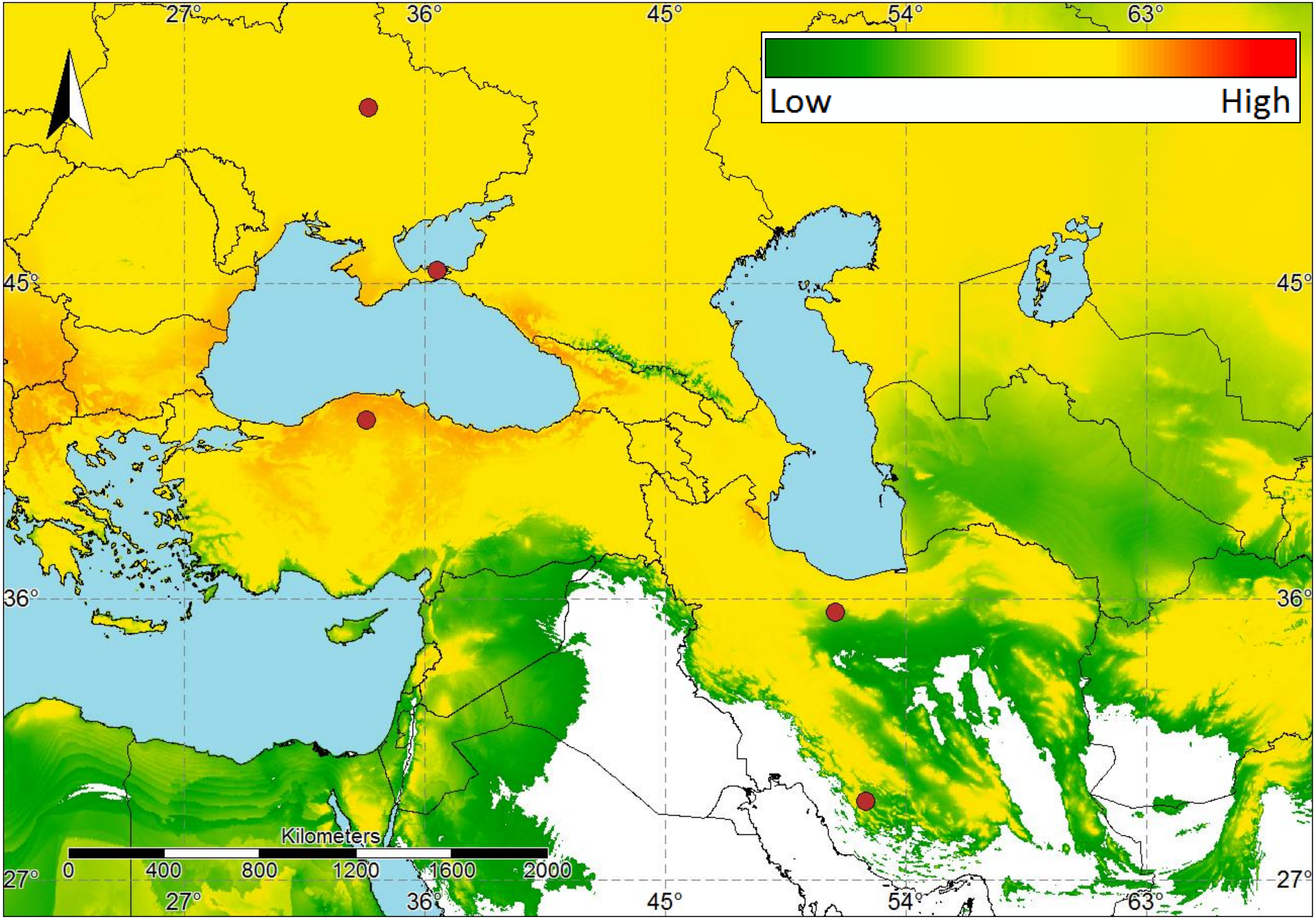

27°
36°
45°
54°
63°
45°
36°
27°
Low
High
Kilometers
0
400
800
1200
1600
2000

**(b)**

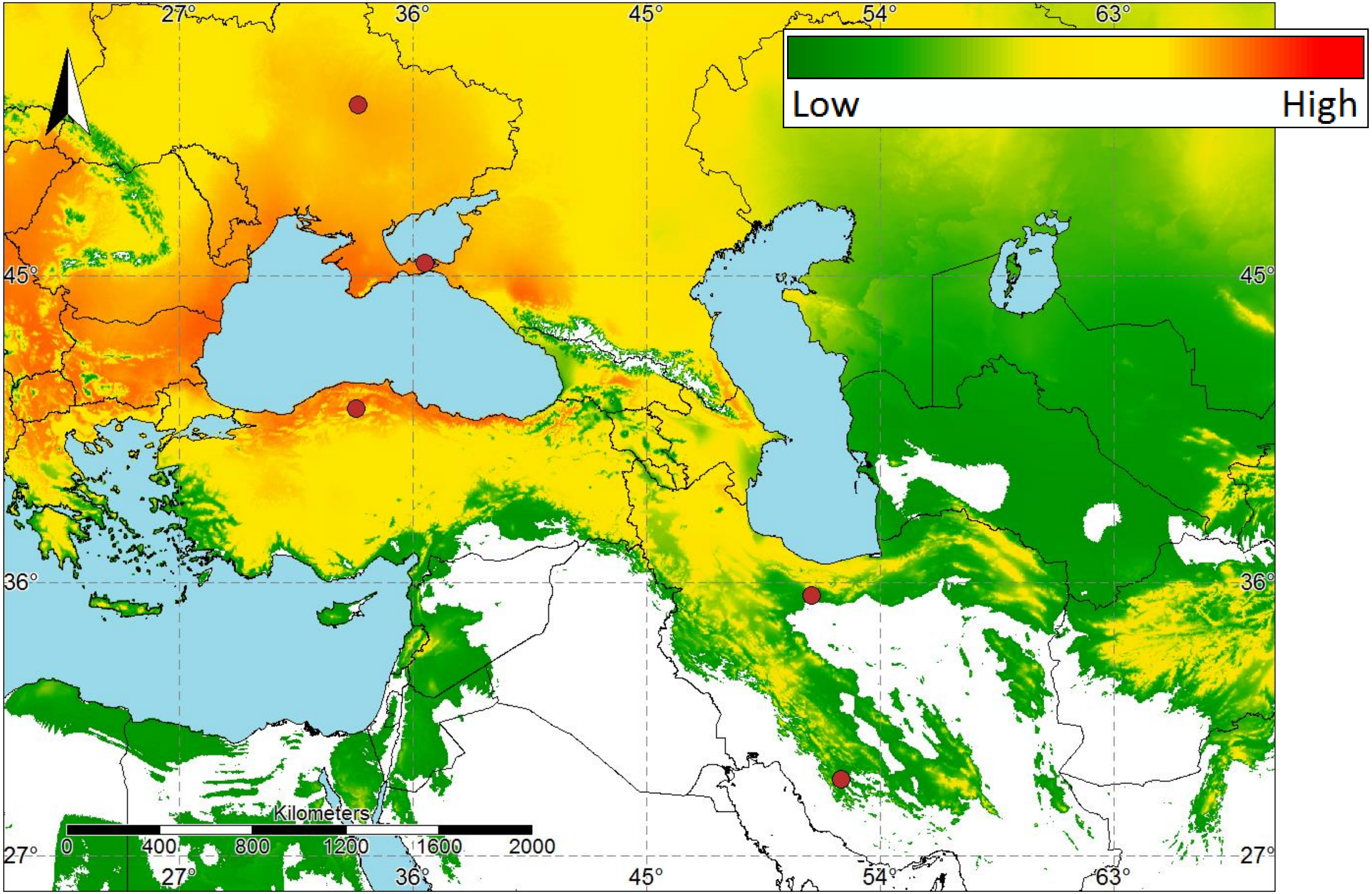


**Figure S1.10.** Detailed maps of potentially useful niche for *Ch. stipae stipae* in the Caucasus and Middle East with its known distribution. (a) Model based only on climate variables, and (b) model based on climate variables and outcome model of potential distribution of *Stipa* species. Maps were plotted using SAGA GIS 3.0.0[1] (http://www.saga-gis.org); projection – World Mercator (EPSG: 3395).

(a)

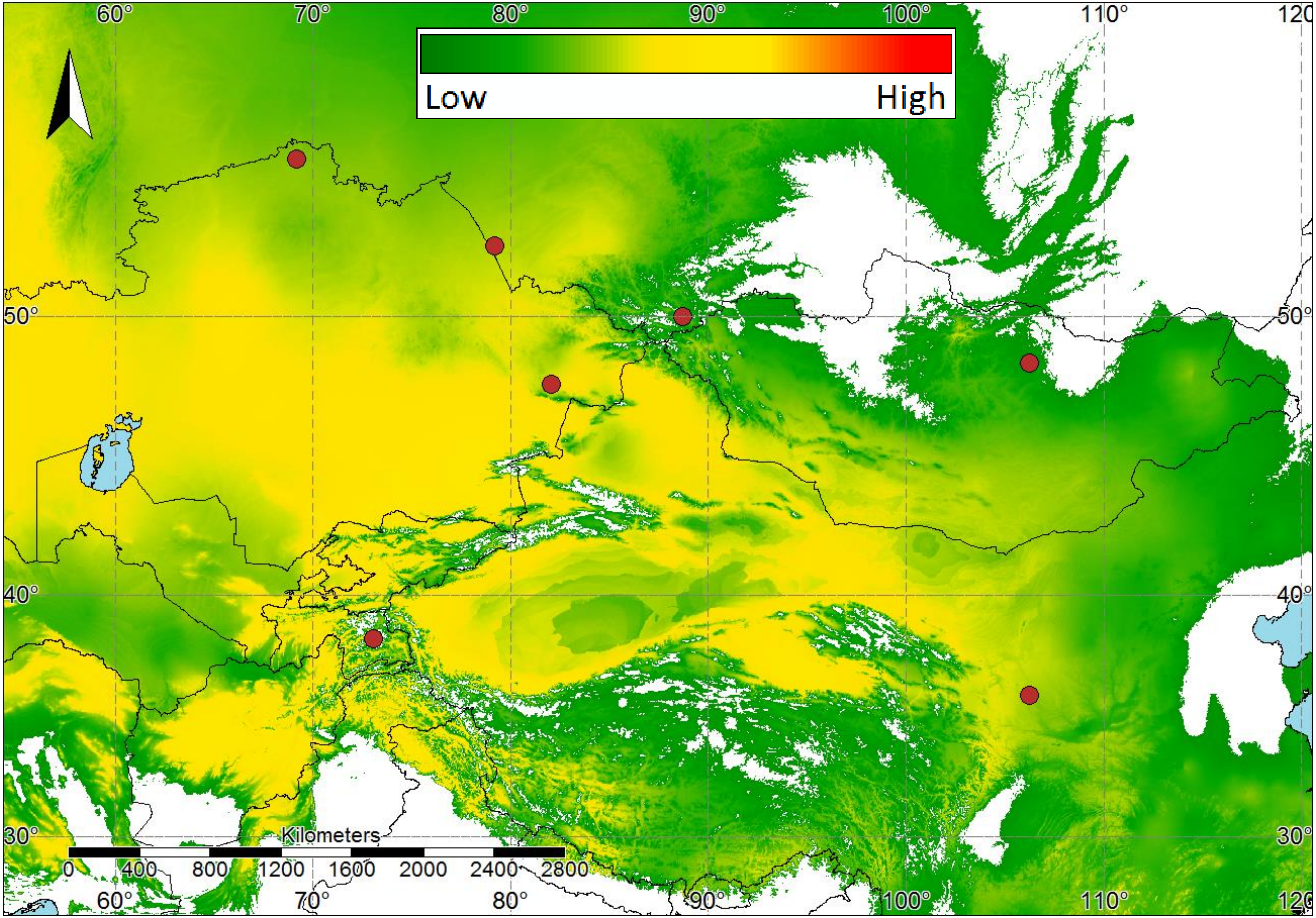
Low
High
Kilometers
0
400
800
1200
1600
2000
2400
2800
60°
70°
80°
90°
100°
110°
120
50°
40°
30°

(b)

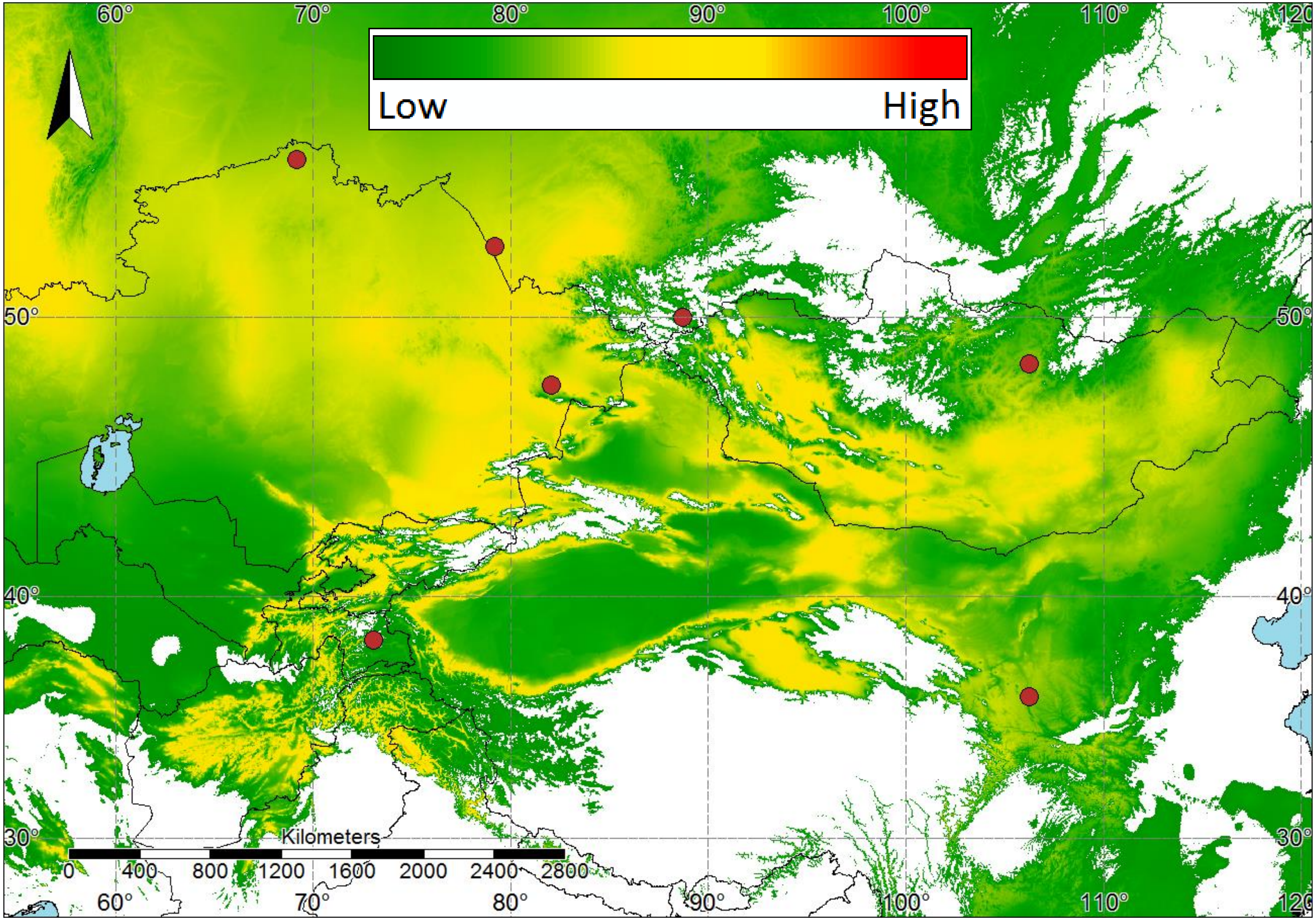


**Figure S1.11.** Detailed maps of potentially useful niche for *Ch. stipae stipae* in Asia (mainly Kazakhstan, Mongolia and China) with its known distribution. (a) Model based only on climate variables, and (b) model based on climate variables and outcome model of potential distribution of *Stipa* species. Maps were plotted using SAGA GIS 3.0.0[1] (http://www.saga-gis.org); projection – World Mercator (EPSG: 3395).

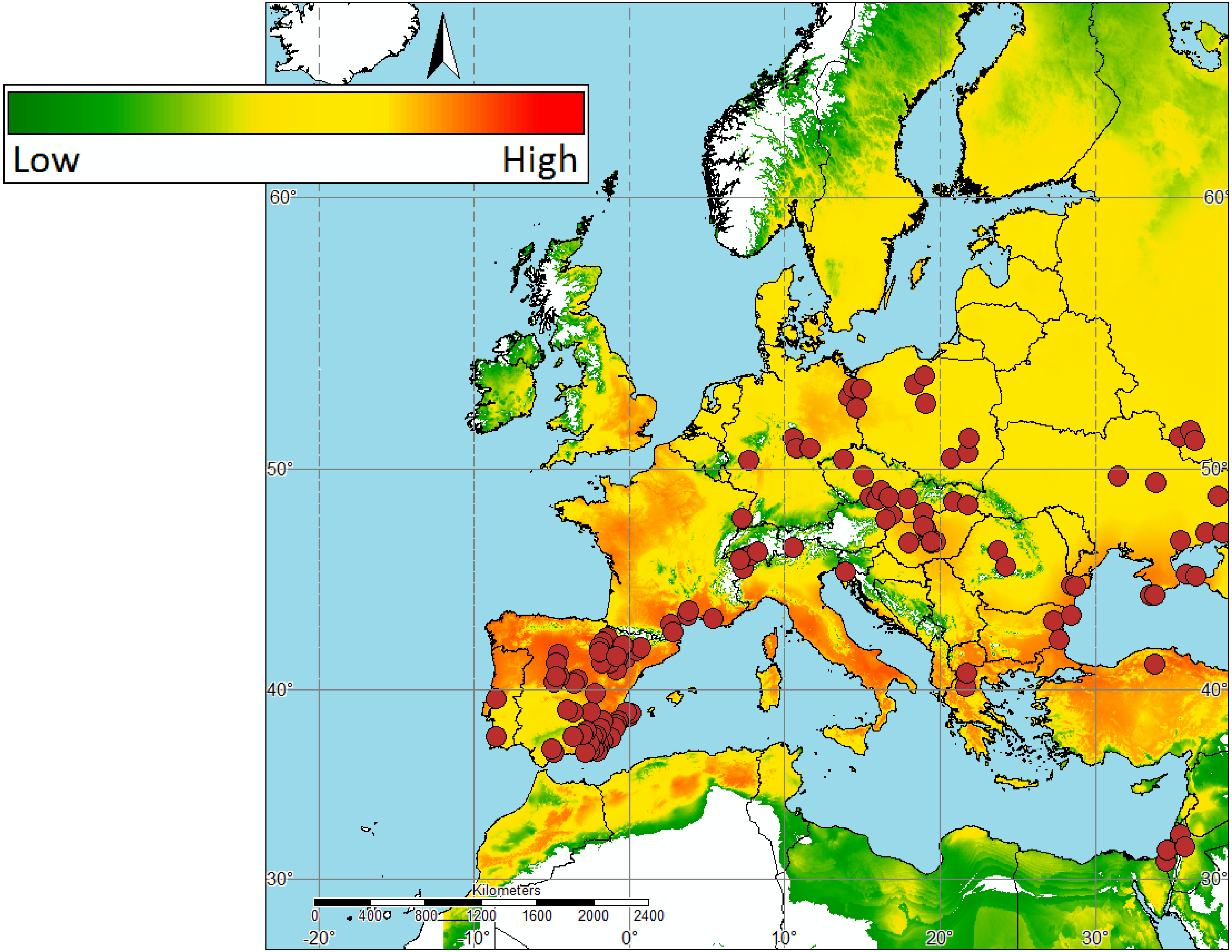


**Figure S1.12.** Detailed maps of potentially useful niche for *Stipa* species in Europe with its known distribution. Map was plotted using SAGA GIS 3.0.0[1] (http://www.saga-gis.org); projection – World Mercator (EPSG: 3395).

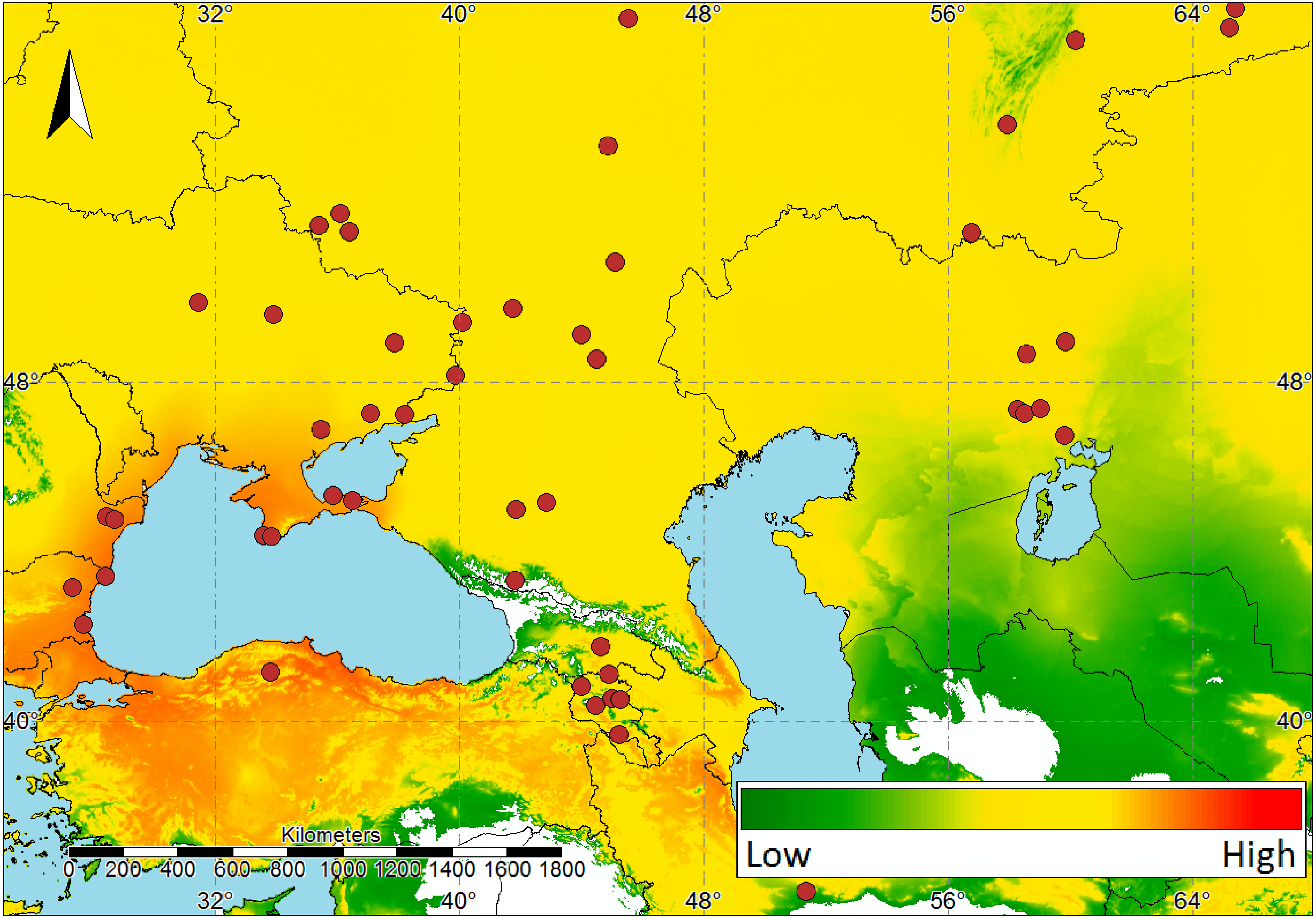


**Figure S1.13.** Detailed maps of potentially useful niche for *Stipa* species in the Caucasus, Russia and Kazakhstan with its known distribution. Map was plotted using SAGA GIS 3.0.0[1] (http://www.saga-gis.org); projection – World Mercator (EPSG: 3395).

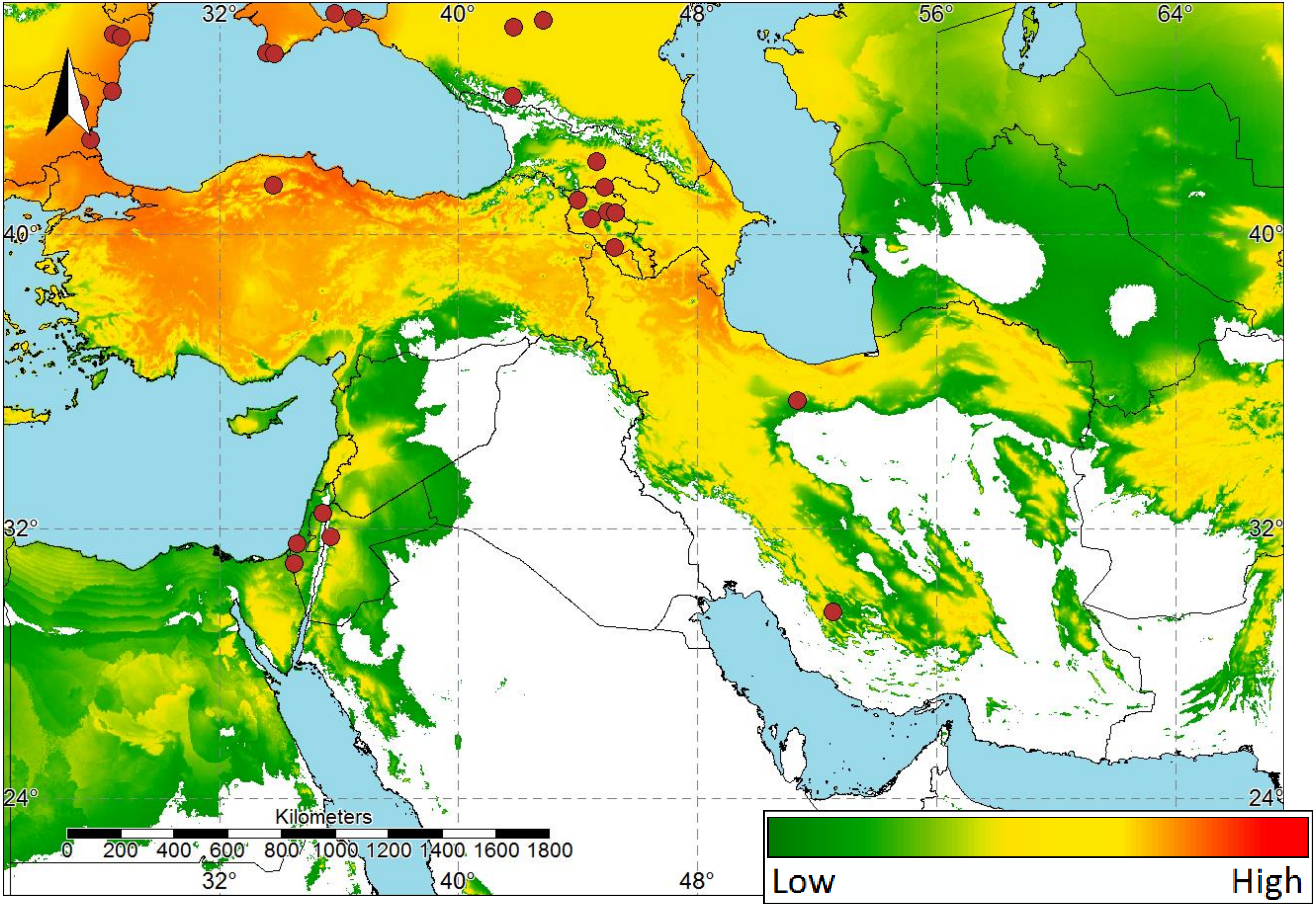


**Figure S1.14.** Detailed maps of potentially useful niche for *Stipa* species in the Caucasus and Middle East with its known distribution. Map was plotted using SAGA GIS 3.0.0[1] (http://www.saga-gis.org); projection – World Mercator (EPSG: 3395).

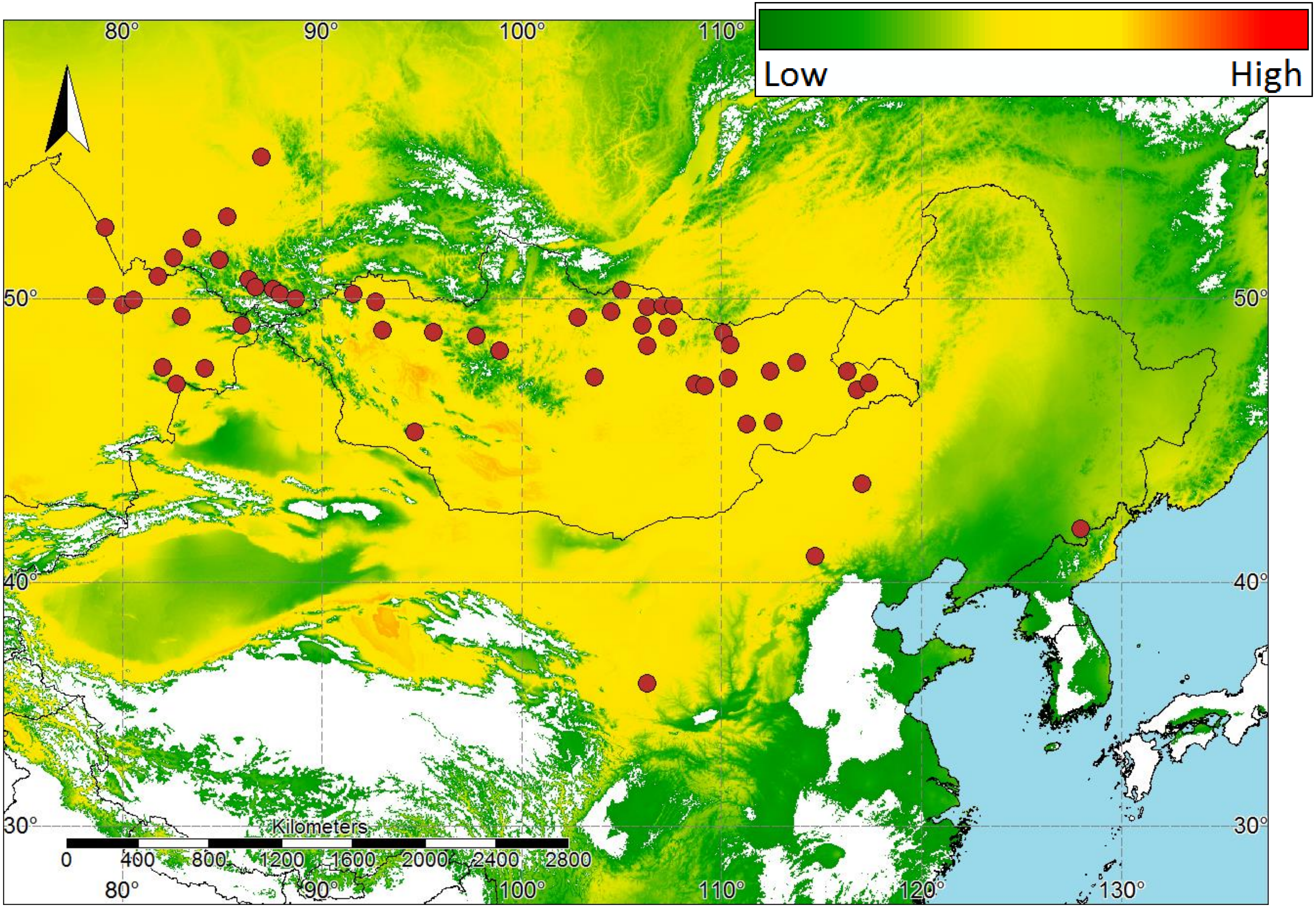


**Figure S1.15.** Detailed maps of potentially useful niche for *Stipa* species in Asia (mainly Kazakhstan, Russia, Mongolia and China) with its known distribution. Map was plotted using SAGA GIS 3.0.0[1] (http://www.saga-gis.org); projection – World Mercator (EPSG: 3395).

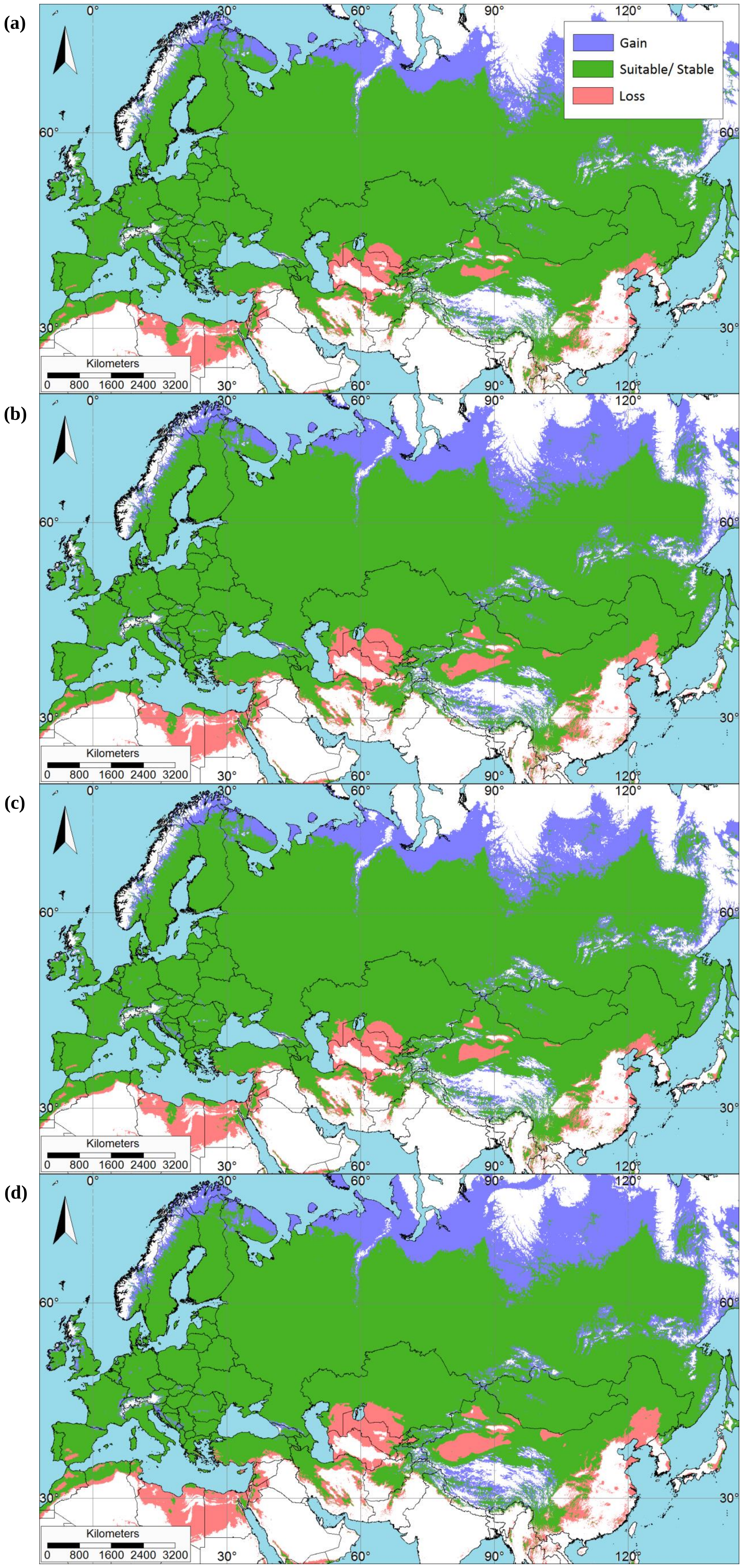


**Figure S1.16.** Predicted future (unsuitable, suitable/stable, lost and gained) habitat for *Stipa* species. Future predictions are based on an ensemble of predictions under all four RCP scenarios ((a) +2.5, (b) +4.5, (c) +6.0 and (d) +8.5 W/m$^2$) for 2050s (>10$^{th}$ percentile training presence threshold). The map was plotted using SAGA GIS 3.0.060 (http://www.saga-gis.org); projection – World Mercator (EPSG: 3395).

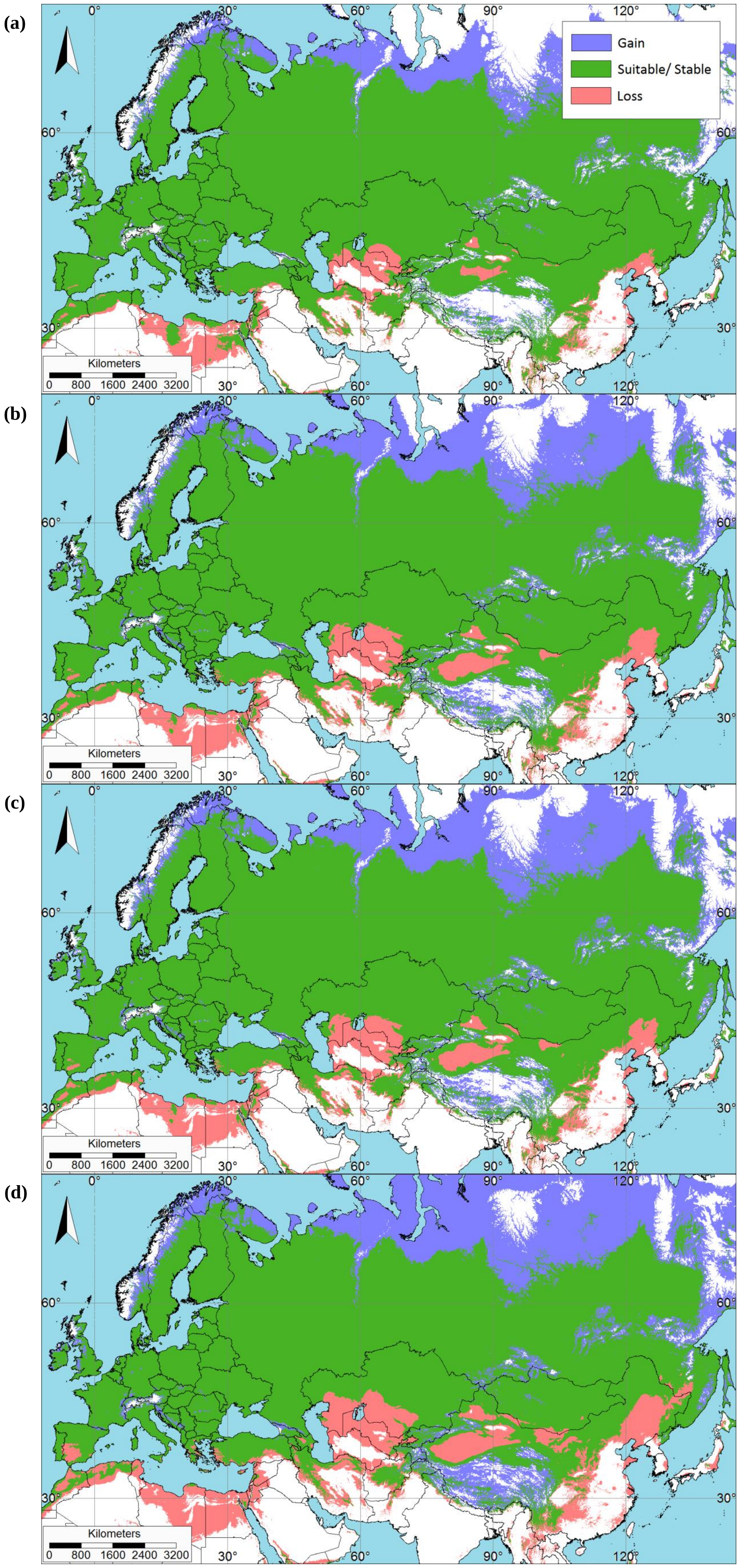


**Figure S1.17.** Predicted future habitat for *Stipa* species. Future predictions are based on an ensemble of predictions under all four RCP scenarios ((a) +2.5, (b) +4.5, (c) +6.0 and (d) +8.5 W/m$^2$) for 2070s (>10$^{th}$ percentile training presence threshold). The map was plotted using SAGA GIS 3.0.060 (http://www.saga-gis.org); projection – World Mercator (EPSG: 3395).

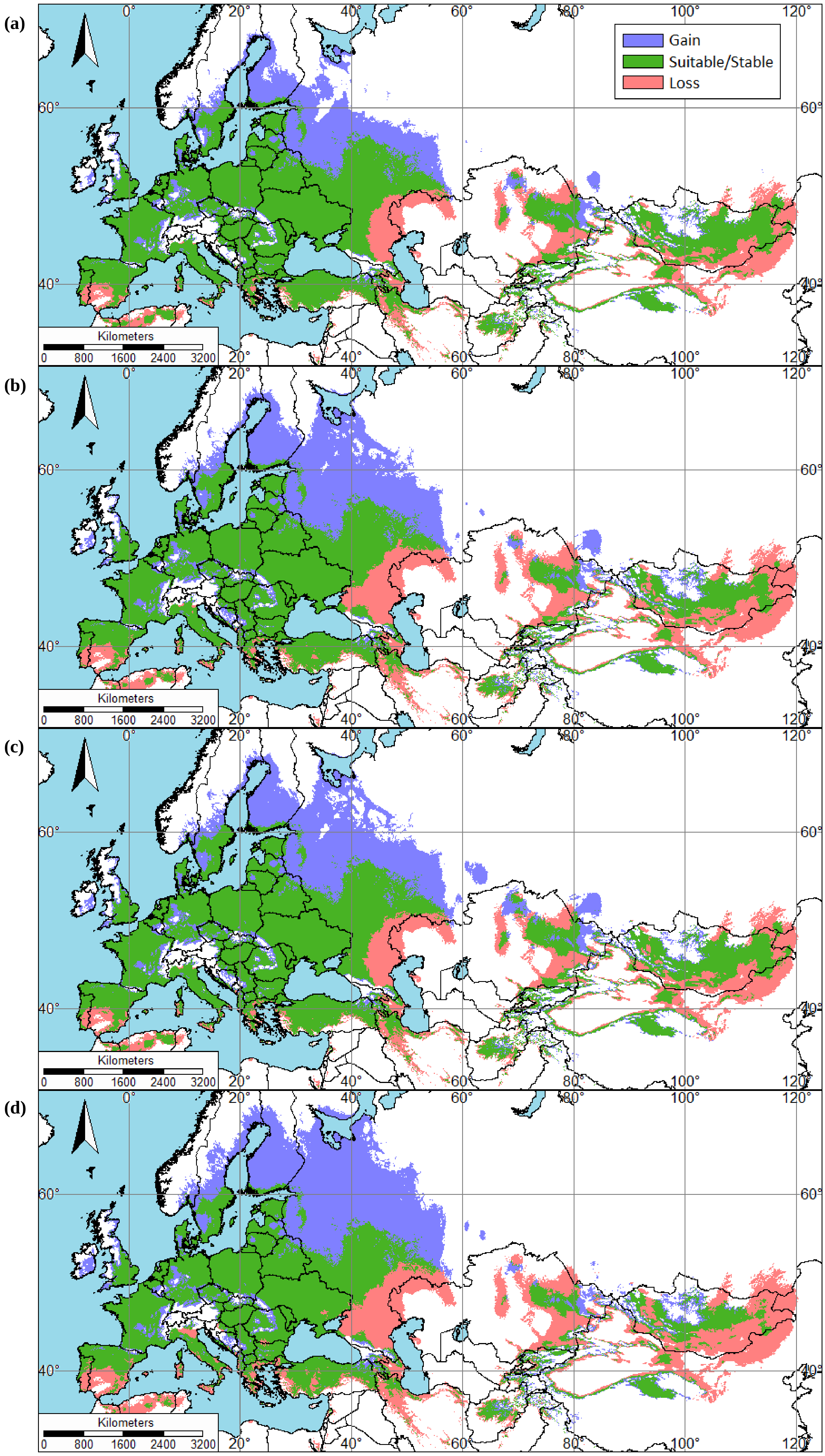


**Figure S1.18.** Predicted future habitat for *Stipa* species. Future predictions are based on an ensemble of predictions under all four RCP scenarios ((a) +2.5, (b) +4.5, (c) +6.0 and (d) +8.5 W/m$^2$) for 2050s (>50$^{th}$ percentile training presence threshold). The map was plotted using SAGA GIS 3.0.060 (http://www.saga-gis.org); projection – World Mercator (EPSG: 3395).

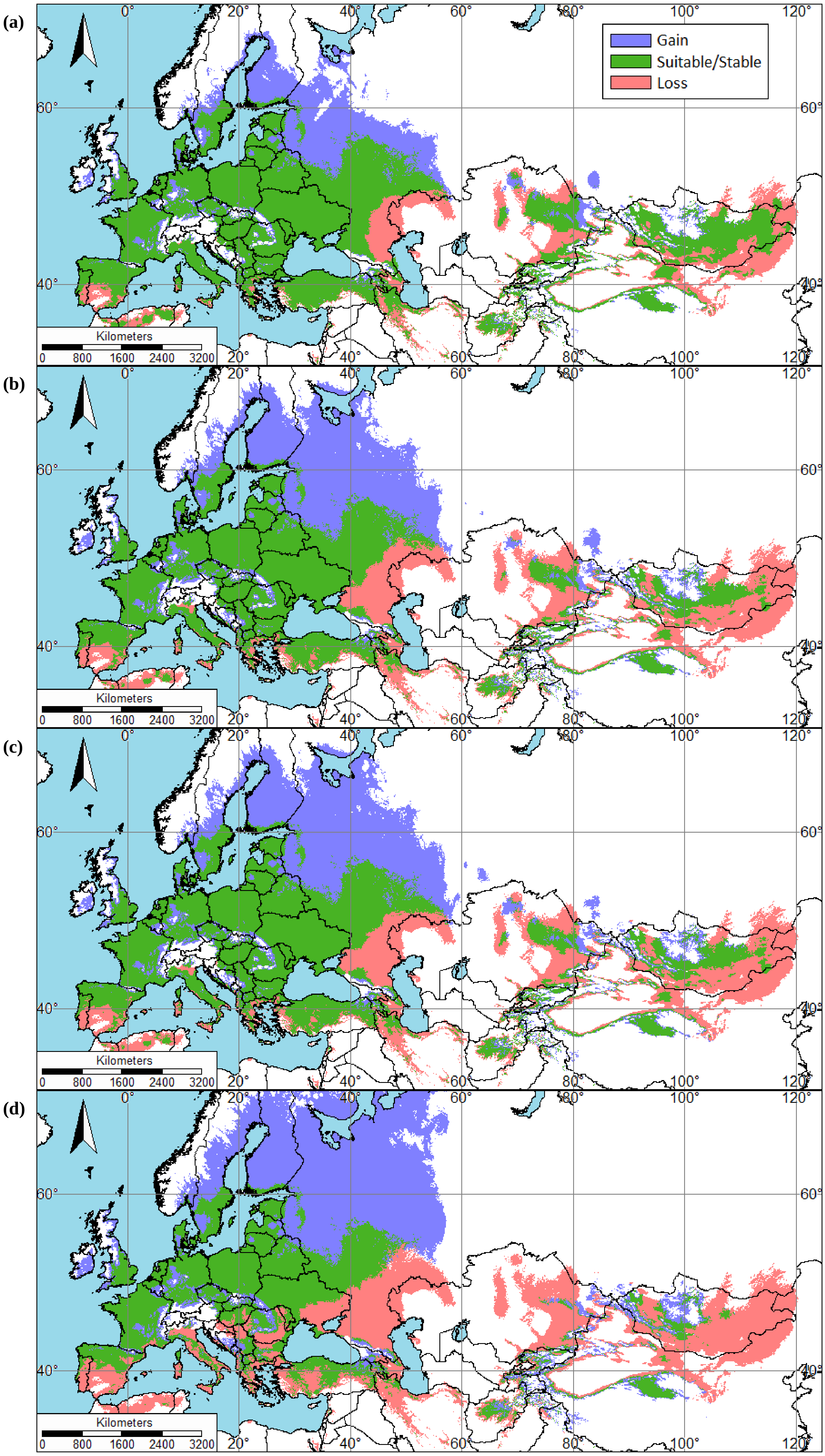


**Figure S1.19.** Predicted future habitat for *Stipa* species based on an ensemble of predictions under all four RCP scenarios ((a) +2.5, (b) +4.5, (c) +6.0 and (d) +8.5 W/m$^2$) for 2070s (>50$^{th}$ percentile training presence threshold). The map was plotted using SAGA GIS 3.0.0[60] (http://www.saga-gis.org); projection – World Mercator (EPSG: 3395).

(a)

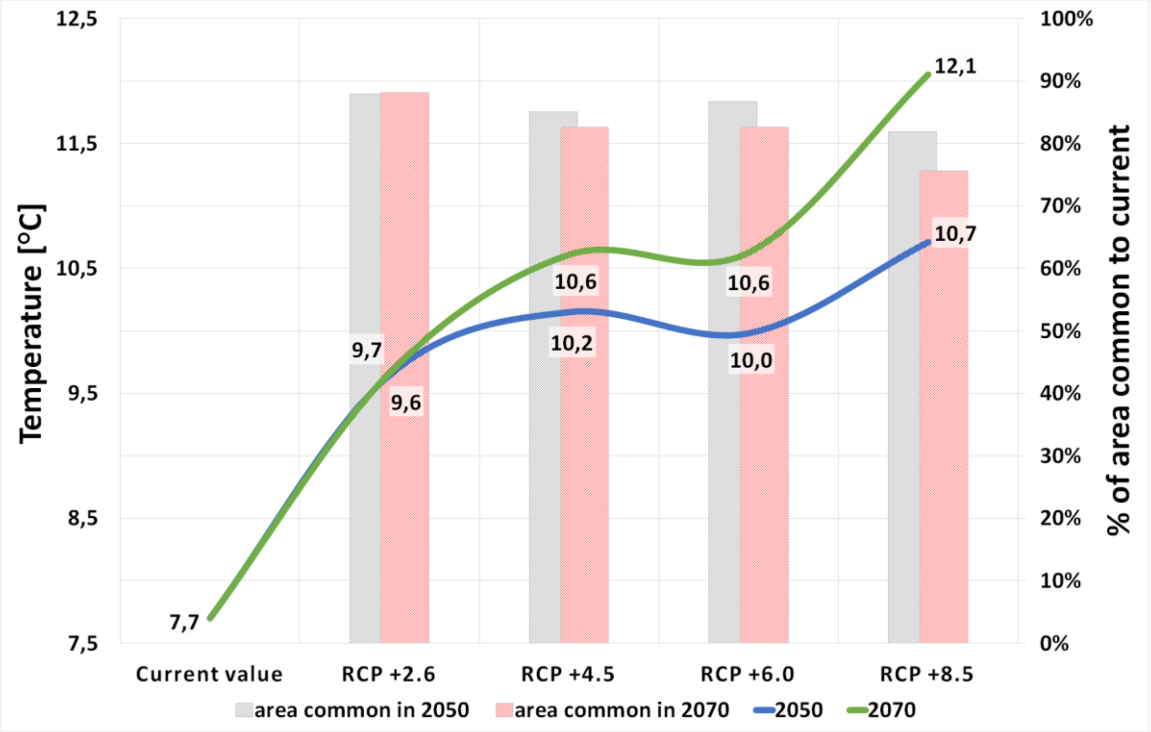


(b)

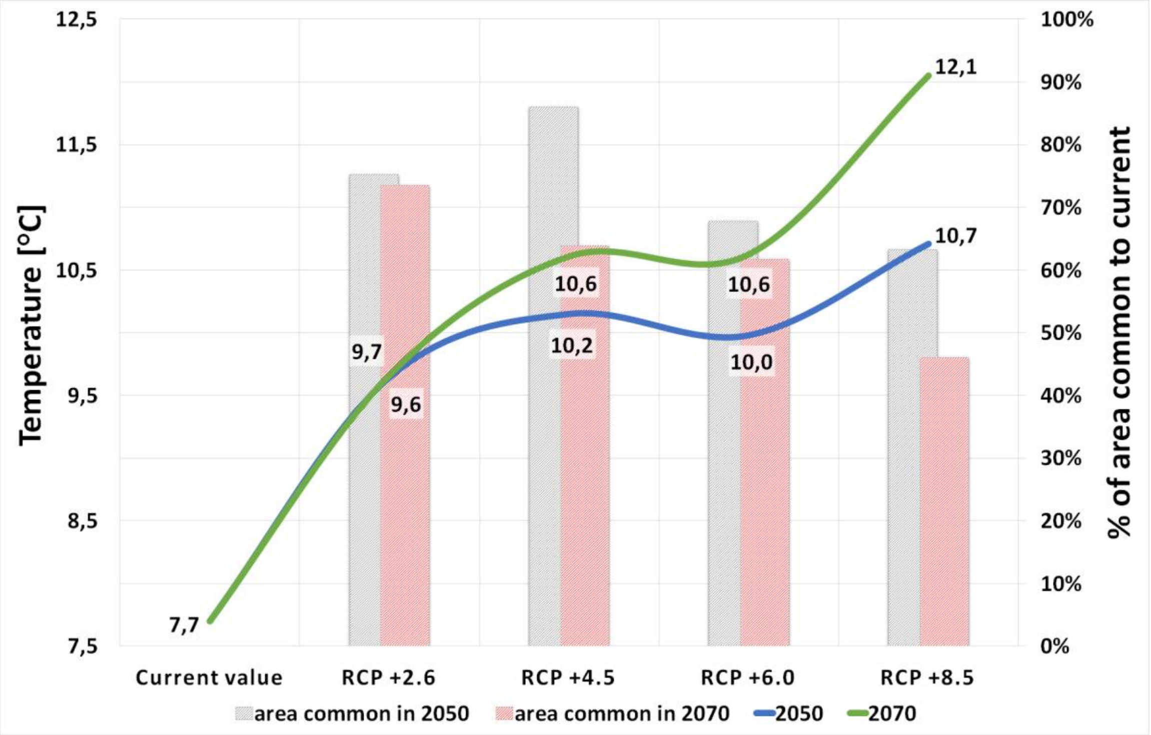


**Figure S1.20.** The average increase of mean annual temperature, under all four RCP scenarios for 2050s and 2070s, in areas where representatives of grasses of the genus *Stipa* are currently presented. Graph also presents percent of area common to current under climate change scenarios, based on modeling results (a) for 10$^{th}$ percentile training presence threshold, and (b) for 50$^{th}$ percentile training presence threshold (see Table 1 in the main text).

References:

1. Conrad, O. *et al*. System for Automated Geoscientific Analyses (SAGA) v. 2.2.7. *Geosci. Model Dev*. **8**, 1991–2007 (2016).
